# Ligand-Aided Glycolysis of PET Using Functionalized Silica-Supported Fe_2_O_3_ Nanoparticles

**DOI:** 10.1021/acssuschemeng.3c03585

**Published:** 2023-10-18

**Authors:** Éadaoin Casey, Rachel Breen, Jennifer S. Gómez, Arno P. M. Kentgens, Gerard Pareras, Albert Rimola, Justin. D. Holmes, Gillian Collins

**Affiliations:** † School of Chemistry, University College Cork, Cork T12 YN60, Ireland; ‡ AMBER Centre, Environmental Research Institute, University College Cork, Cork T23 XE10, Ireland; § Institute for Molecules and Materials, Radboud University, Nijmegen 6525 AJ, The Netherlands; ∥ Departament de Química, Universitat Autònoma de Barcelona, Bellaterra, Catalonia 08193, Spain

**Keywords:** heterogeneous catalysts, glycolysis, SiO_2_, nanoparticles, polyethylene terephthalate, DFT, solid-state NMR

## Abstract

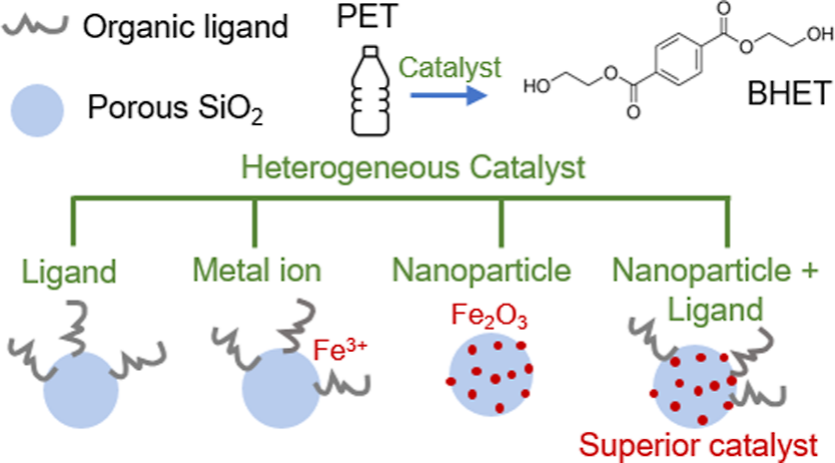

The development of efficient catalysts for the chemical recycling of poly(ethylene terephthalate) (PET) is essential to tackling the global issue of plastic waste. There has been intense interest in heterogeneous catalysts as a sustainable catalyst system for PET depolymerization, having the advantage of easy separation and reuse after the reaction. In this work, we explore heterogeneous catalyst design by comparing metal-ion (Fe^3+^) and metal-oxide nanoparticle (Fe_2_O_3_ NP) catalysts immobilized on mesoporous silica (SiO_2_) functionalized with different N-containing amine ligands. Quantitative solid-state nuclear magnetic resonance (NMR) spectroscopy confirms successful grafting and elucidates the bonding mode of the organic ligands on the SiO_2_ surface. The surface amine ligands act as organocatalysts, enhancing the catalytic activity of the active metal species. The Fe_2_O_3_ NP catalysts in the presence of organic ligands outperform bare Fe_2_O_3_ NPs, Fe^3+^-ion-immobilized catalysts and homogeneous FeCl_3_ salts, with equivalent Fe loading. X-ray photoelectron spectroscopy analysis indicates charge transfer between the amine ligands and Fe_2_O_3_ NPs and the electron-donating ability of the N groups and hydrogen bonding may also play a role in the higher performance of the amine-ligand-assisted Fe_2_O_3_ NP catalysts. Density functional theory (DFT) calculations also reveal that the reactivity of the ion-immobilized catalysts is strongly correlated to the ligand–metal binding energy and that the products in the glycolysis reaction catalyzed by the NP catalysts are stabilized, showing a significant exergonic character compared to single ion-immobilized Fe^3+^ ions.

## Abstract 2

Low metal loading catalysts for PET glycolysis using surface-bound organic amines to enhance the activity of Fe_2_O_3_ NPs on SiO_2_ support.

## 1 Introduction

In recent years, research in developing pathways to convert waste polymers into its constituent monomers or value-added products has grown exponentially with the key goal of achieving a circular economy in the polymer life cycle.^1^ Polyethylene terephthalate (PET) is a very useful polymer in our everyday life which includes textiles, packaging, beverages among many others.^2,3^ However, it is also one of the largest components of postconsumer plastic waste in landfills, highlighting the need for effective recycling strategies for this polymer. This has gained huge interest for researchers to find low cost, and environmental friendly ways of degrading PET to useful monomers with the aim of achieving a circular economy in the polymer life cycle.^2,4^ There are a variety of chemical recycling pathways for PET including, hydrolysis,^5^ alcoholysis,^6^ and glycolysis.^3,7^ Glycolysis is particularly attractive due to its low cost and mild reaction conditions compared to other depolymerization pathways.^8^ The process involves transesterification reaction of PET with excess glycol, usually ethylene glycol, to obtain bis(hydroxyethyl) terephthalate (BHET).^9^ BHET can be repolymerized to PET and it is also used in the synthesis of unsaturated polyesters, rigid or flexible polyurethanes, and other fine chemicals.^10^

The glycolysis of PET is typically performed in the presence of a catalyst as the reaction is slow and requires elevated temperatures in the absence of a catalyst.^11^ Homogeneous catalysts such as metal salts,^12^ metal oxides,^13^ and ionic liquids^2,14^ are the most commonly used; however, this leads to issues regarding catalyst recovery and contamination of the BHET product.

Numerous heterogeneous catalysts have been developed for PET glycolysis, which are advantageous compared to homogeneous catalysts because they can be easily recovered and reused. Biomass-derived heterogeneous catalysts have been prepared from waste orange peel ash,^15^ waste bamboo leaf ash, and calcium-oxide-based catalysts made from eggshells and seafood shells.^16^ Cobalt nanoparticles (3 nm) stabilized by tannic acid ligands showed high activity for PET glycolysis with 96% conversion and 77% BHET yield.^17^ Various metal oxides nanostructures such as ZnO^18^ and Fe_3_O_4_^19^ nanodispersions have been reported. Son et al.^20^ reported the use of exfoliated manganese oxide nanosheets for PET glycolysis, leading to full conversion of PET at 0.01 wt % catalysts, at 200 °C after 30 min. Molybdenum-doped ultrathin ZnO nanosheets display far superior yields of BHET (94.5%) than standard undoped ZnO catalysts (54.7%) which have been previously reported in literature.^21^ The Mo atoms replace Zn atoms at defect sites, forming Mo–Zn bonds, influencing the electronic structure of the catalyst, and promoting electron transfer of the glycolysis reaction. Mesoporous SBA-15 catalysts doped with ZnO gave a 91% BHET yield, and the catalyst displayed good stability and high catalytic activity when recycled. Ion-based heterogeneous catalysts such as metal organic frameworks (MOFs)^22,23^ and zeolites^24^ have also been reported as good catalysts for the depolymerization of PET due to their highly ordered structures, high surface areas, and porosity.^23^ Yang et al.^25^ reported the use of metal azolate framework-6 catalyst (a sub-class of MOFs) with a high density of zinc ion species immobilized on the surface achieving 92.4% conversion of PET and 81.7% yield of BHET. Wang et al.^26^ investigated the use of metal ions immobilized on a polymer ionic liquid, (BVim)NTf_2_-Zn^2+^, which gave 95.4% PET conversion and 77.8% BHET yield.

**Scheme 1:**
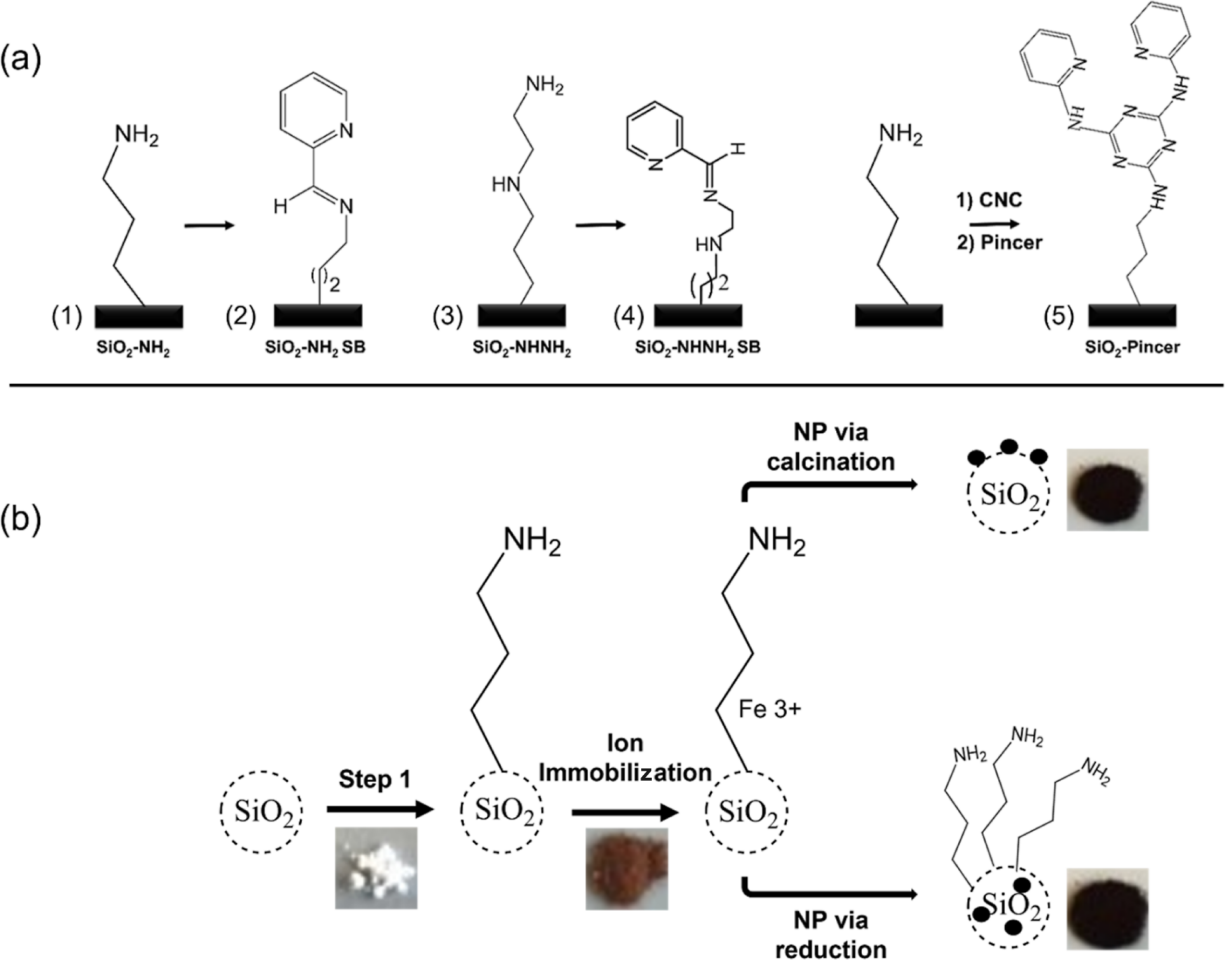
(a) Synthesis Pathways to Prepare Mesoporous SiO_2_ with Five Different Organic Capping Ligands SiO_2_–NH_2_ (1); SiO_2_–NH_2_–SB (2); SiO_2_–NH–NH_2_ (3), SiO_2_–NH–NH_2_–SB (4) and SiO_2_–Pincer (5), and (b) the Catalysts Preparation Scheme

It is evident from the literature that a wide variety of heterogeneous catalysts are effective for PET Glycolysis, ranging from ion- immobilized catalysts to nanoparticles, and Table S1 summarizes reports of heterogeneous catalysts and conditions in the literature. The aim of this work is to gain insight into the optimum heterogeneous catalyst design for PET glycolysis with a particular focus on the immobilization of metal-ion and nanoparticle-based catalysts. Surface modification of a catalyst support, such as SiO_2_, with organic linkers is a convenient strategy to immobilize a high density of transition metal ions dispersed on a support material. The nature of the organic linker used to tether the ion can influence the steric and the electronic environment with the metal center, which in turn can influence catalytic activity.^27^ In this work, Fe ions were immobilized onto porous silica functionalized with different organic linkers, which are illustrated in Scheme 1. Solid-state NMR is used for quantitative analysis of the modified surfaces. The ion-based catalysts were then converted to NP catalysts either by calcination, which removes the organic ligands, or by chemical reduction, which preserves the ligand. The heterogeneous catalysts were evaluated for PET glycolysis to gain insight into how the immobilization of the metal species and the organic ligands influenced catalytic performance. Finally, DFT calculations were carried out to unveil the reaction mechanism, and by this way correlating experimental observations with an atomistic interpretation.

## 2 Results and Discussion

### 2.1 Catalyst Design and Synthesis

Silica is an excellent catalytic support material that allows the surface chemistry to be easily altered through surface functionalization using silylation chemistry.^28^ Mesoporous cellular foam (MCF) SiO_2_ is a three-dimensional (3D)-connected pore structure that was chosen in this study because of its relatively large pore diameter (100 nm) in comparison to those of other porous silicas. Scheme 1a shows the synthesis pathways to prepare mesoporous SiO_2_ with five different capping ligands: SiO_2_–NH_2_ (1), SiO_2_–NH_2_–SB (2), SiO_2_–NH–NH_2_ (3), SiO_2_–NH–NH_2_–SB (4), and SiO_2_–pincer (5) (see the Supporting Information for Experimental Section and reaction details).

**Figure 1:**
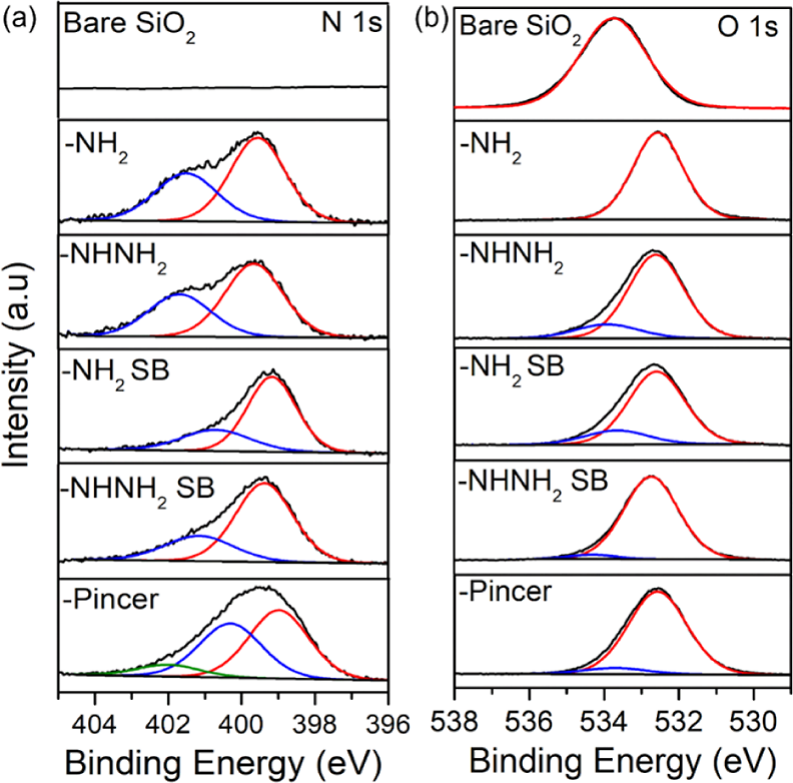
XPS analysis of the (a) N 1s spectra and (b) O 1s core level for bare silica and the functionalized SiO_2_ with –NH_2_ (1), –NH_2_ SB (2), –NHNH_2_ (3), –NHNH_2_ SB (4), and pincer (5), respectively.

X-ray photoelectron spectroscopy (XPS) was employed to verify the successful functionalization of the SiO_2_ support with each of the five ligands. After functionalization, a N signal appeared in all the N 1s core levels as shown in Figure 1a. All catalysts displayed a dominant peak at a binding energy (BE) of 399 eV attributed to the amine functional group and a shoulder peak at ∼400 to 402 eV attributed to protonated amines typically observed on modified SiO_2_.^29^ No N signal was detected in bare SiO_2_. The O 1s core level, shown in Figure 1b, also confirms functionalization with the BE of the bare silica centered at 533.75 eV downshifted to 532.5 eV, associated with the loss of Si–OH groups after surface modification.^30^ The Si 2p core level of bare SiO_2_, shown in the Supporting Information Figure S2, shows a single peak at 104.25 eV and after functionalization, the Si 2p peak can be deconvoluted in several peaks associated with different Si environments.

**Figure 2:**
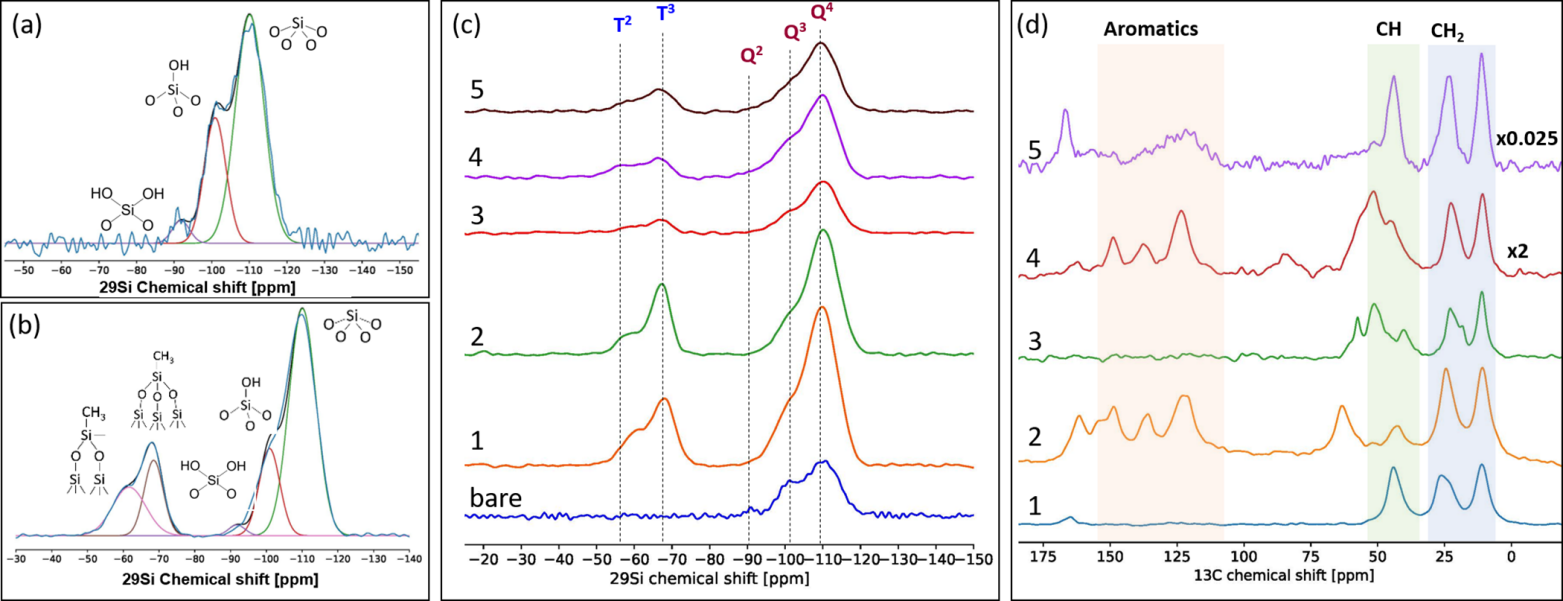
1D (a) ^29^Si MAS NMR spectra together with their deconvolution of bare silica and (b) functionalized SiO_2_–NH_2_ (1). (c) Stacked plot of the ^29^Si MAS NMR spectra of all samples; bare silica, SiO_2_–NH_2_ (1), SiO_2_–NH_2_–SB (2), SiO_2_–NH–NH_2_ (3), SiO_2_–NH–NH_2_–SB (4), and SiO_2_–pincer (5); (d) ^1^H → ^13^C CPMAS NMR spectra of the functionalized silica with ligands (1) to (5).

It is well recognized that silylation chemistry can result in significantly more complicated surface attachment chemistry than is often illustrated across the literature.^29^ Solid-state NMR is a powerful tool to provide insight into the grafting chemistry.^31,32^ The 1D ^29^Si and ^13^C NMR spectra of the bare and five different functionalized SiO_2_ ions are presented in Figure 2. For the ^29^Si NMR spectra, spectral deconvolution was carried out using ssNake^33^ (using Gaussian/Lorentzian line-shapes for fitting) to identify the individual species present. The ^29^Si NMR spectrum of bare SiO_2_ (Figure 2a) exhibit resonances at ca. −91.8, −100.9, and −110.1 ppm, corresponding to *Q*^2^, *Q*^3^, and *Q*^4^ sites, respectively. Detailed fitting of these spectra is shown in Figure 2a,b. The spectra of all six samples in Figure 2c, show the presence of Si(SiO)_*n*_(OH)_4–*n*_ sites (denoted *Q*^*n*^ with *n* = 2, 3 and 4), characteristic of silica-type materials.^34^

Figure 2c compares the NMR spectra of unfunctionalized and functionalized SiO_2_. The spectra of the functionalized SiO_2_ show the appearance of two peaks centered at −67.3 and −60 ppm, which arise from T^2^ [(≡SiO−)_2_SiR(−OH)], with R being the alkyl chain)) and T^3^ [(≡SiO−)_3_SiR] sites, respectively,^32,35^ confirming the successful grafting of the amine groups onto SiO_2_.

Quantitative ^29^Si NMR spectra allowed the determination of the relative surface coverage of each species present (*Q*^*n*^ and *T*^*n*^ sites) (see Table 1). The relative areas of *Q*^4^ sites remains almost constant in all the samples, and the degree of condensation of the Si atoms as calculated in Table 1 remain almost constant for all the ligands, confirming that the SiO_2_ structure remains intact after functionalization. In addition, the low content of *Q*^2^ sites indicates the preference of the ligands to occupy the *Q*^2^ sites. The relative areas of *Q*^*n*^ sites can be used to determine the degree of surface functionalization, giving surface coverages between 40 and 73%, as shown in Table 1. However, no clear correlation between the surface coverage and structure of the ligand was found, but the analysis demonstrates that the attachment of the ligand, and therefore coverage, is strongly influenced by the primary linker molecule. When the Schiff base (SB) ligand is reacted with SiO_2_–NH_2_ to convert ligand 1 to ligand 2 there is a decrease in *Q*^2^ + *Q*^3^ sites (with increasing *T*^3^ + *T*^2^), indicating that the SB precursor (2-pyridinecarboxaldehyde) is attaching to surface –OH groups of the SiO_2_ particles in addition to the terminal –NH_2_ groups. Consequently, the SB ligand has a significantly higher surface coverage (73%) compared to those of the other ligands. This statement is supported by the appearance of an additional peak in the ^13^C CPMAS spectra of the SiO_2_–NH_2_ SB and SiO_2_–NH–NH_2_ SB catalysts (in the region of 50–100 ppm) (Figure S3). This signal could be attributed to the presence of an aldehyde group^36^ with the surface silanol groups, which would explain the differences observed in ^29^Si NMR. In contrast, using the SiO_2_–NH–NH_2_ ligand (ligand 3 → 4) to attach the SB resulted in an increase in the relative areas for the *Q*^2^ + *Q*^3^. This observation may indicate that the NH_2_ groups in the diamino ligands interact with SiO_2_ surface likely due to the longer ligand length and, therefore, silanol groups can be introduced by rehydroxylation.^37^ Consequently, the surface coverage of the SiO_2_–NH–NH_2_–SB catalyst was the lowest of those of the ligands.

**Table 1:** ^29^Si NMR Quantitative Data for Each of Five Different Capping Ligands SiO_2_–NH_2_ (Ligand 1); SiO_2_–NH_2_–SB (Ligand 2); SiO_2_–NH–NH_2_ (Ligand 3), SiO_2_–NH–NH_2_–SB (Ligand 4), and SiO_2_–Pincer (Ligand 5)

		% Si sites								
	sample	*Q*^4^	*Q*^3^	*Q*^2^	*T*^3^	*T*^2^	*Q*^3^ + *Q*^2^	*T* ^3^ + *T* ^2^	relative surface coverage^a^	degree condens. Si atoms^b^
	SiO_2_	68.2	27.8	4.1			31.8			
Ligand 1	SiO_2_ + NH_2_	58.5	15.1	1.5	15.7	9.1	16.6	24.8	47.7	92.4
Ligand 2	SiO_2_ + NH_2_SB	61.1	8	0.4	12.1	18.4	8.4	30.5	73.6	91.7
Ligand 3	SiO_2_ + NHNH_2_	68.5	15.2	1.4	8.2	6.8	16.6	15.0	48.0	93.3
Ligand 4	SiO_2_ + NHNH_2_SB	65.8	18.5	1.7	4.9	9.1	20.2	13.9	36.5	91.5
Ligand 5	SiO_2_ + Pincer	61.7	18.4	0.6	9.9	9.4	19.0	19.2	40.3	92.0

a The relative surface coverage was determined using the equation .

b The degree of condensation of Si atoms was determined using  (see the Supporting Information for details).

Figure 2 shows the stacked ^13^C CPMAS spectra of the functionalized Si–NH_2_ catalysts with their principal functional groups. The presence of the alkyl groups appearing in SiO_2_–NH_2_ (1) and SiO_2_–NH–NH_2_ (3) catalysts (from 60 to 10 ppm) further confirms successful grafting of both amine ligands onto SiO_2_.^38,39^ The SiO_2_–NH_2_ SB and SiO_2_–NH–NH_2_ SB catalysts, show the presence of aromatic peaks between 175 and 100 ppm confirming successful functionalization with the Schiff base ligands.^39^ The alkyl groups and aromatic peaks were also observed in SiO_2_-pincer when the spectrum was recorded at −40 °C, confirming successful functionalization of the pincer ligand.

After organic functionalization, Fe ions were immobilized onto the SiO_2_ (see the Experimental Section for details). We chose iron as the metal catalyst as iron salts are effective for glycolysis of PET and have lower environmental impact compared with other effective metal-ion catalysts such as Zn^2+^. Inductively coupled plasma-mass spectroscopy (ICP-MS) was used to determine the Fe loading in each of the catalysts. Table S2 (see Supporting Information) displays the quantitative results with the Fe loadings for each ligand, with the highest loading for the simple amine ligand SiO_2_–NH_2_ (4172.8 ppm) and lowest for the SiO_2_–pincer ligand (3904 ppm), which can be expected given the bulky nature of the ligand. The Fe-ion-immobilized catalysts were then converted to iron oxide nanoparticle (NP) catalysts by calcination at 450 °C, which removed the organic ligands on the SiO_2_ surface, or by treatment with aqueous NaBH_4_ as a rapid reducing agent, which preserved the organic ligands, as represented in Scheme 1b.

**Figure 3:**
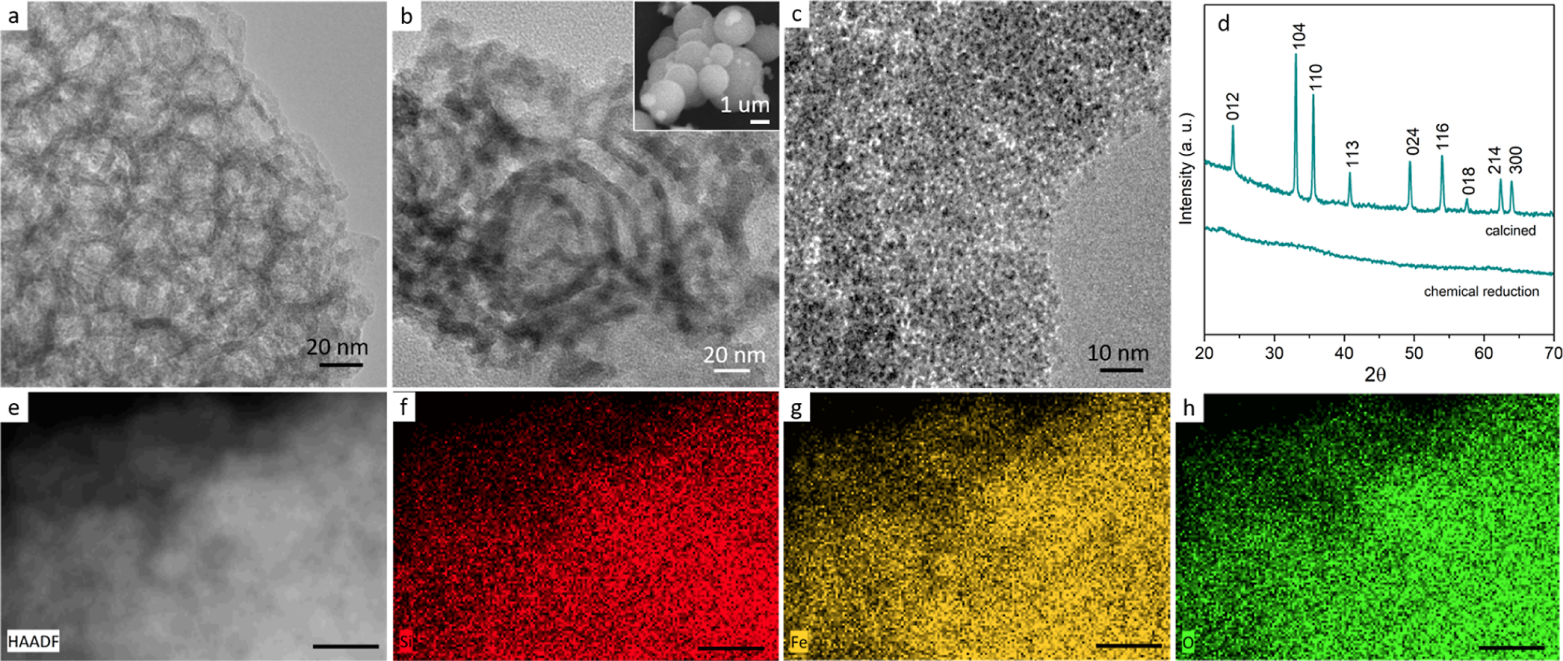
TEM images of SiO_2_ support (a) before Fe-ion immobilized, (b) after calcinations, and (c) after chemical reduction. (d) XRD of SiO_2_ catalysts prepared by calcination and chemical reduction, (e) high angle angular dark field image of SiO_2_ catalyst after chemical reduction, and associated (f) Si, (g) Fe, and (h) O EDX maps. Scale bars in (e,f) are 100 nm.

Figure 3a–c shows the TEM analysis of the SiO_2_ before and after NP formation by calcination and chemical reduction, respectively and associated SEM images are shown in the Supporting Information Figure S4. Catalysts prepared by calcination produced larger NPs compared to catalysts produced by chemical reduction, which is to be expected as rapid reduction by NaBH_4_ is known to produce small diameter NPs.^40^ XRD analysis of the calcined catalysts, shown in Figure 3d, shows the characteristic peaks for α-Fe_2_O_3_ in excellent agreement with JCPDS card no. 33-0664. Diffraction peaks were not observed for the catalyst produced by chemical reduction. Although challenging to visual due to their small size and low atomic mass contrast in TEM, the presence of nanoparticles embedded in the SiO_2_ can be seen in TEM Figure 3c. EDX mapping of the catalyst prepared by chemical reduction is shown in Figure 3e–h and confirms the uniform presence of Fe across the SiO_2_ support.

**Figure 4:**
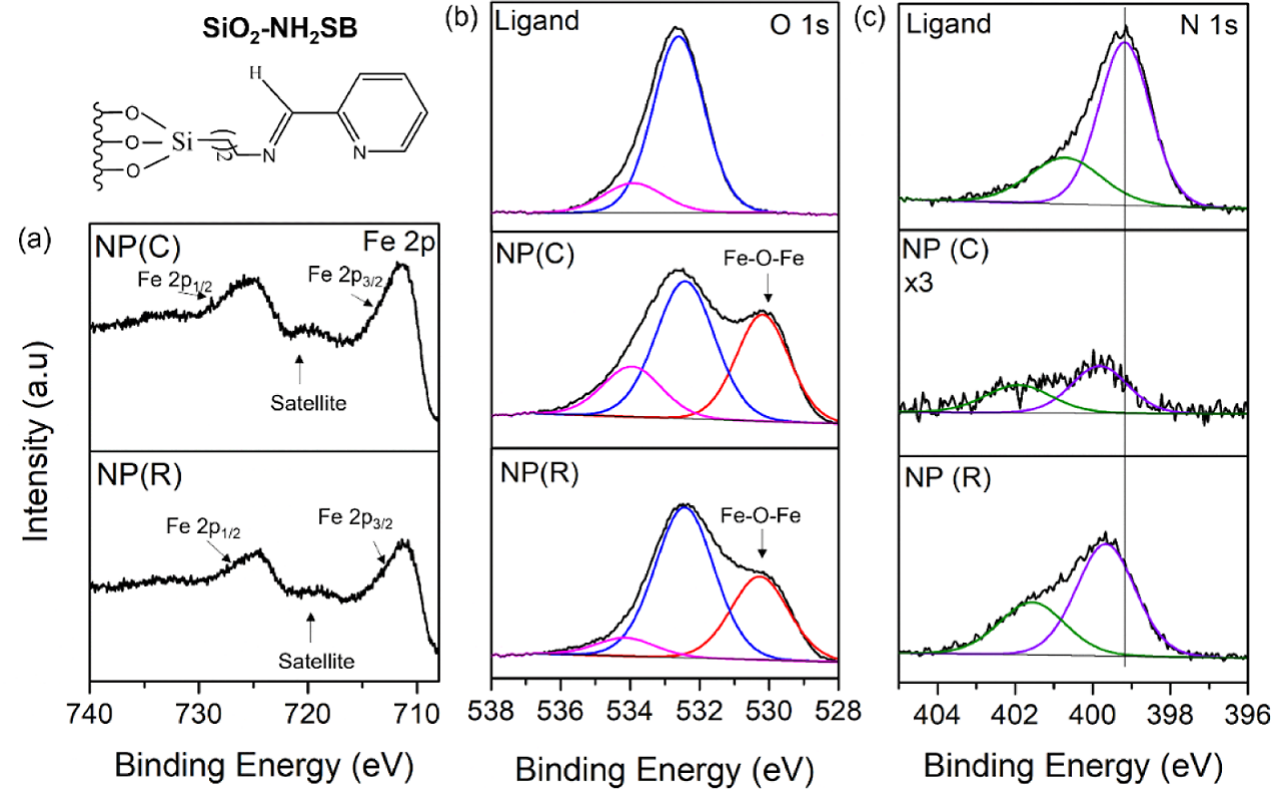
XPS core level spectra of (a) Fe 2p (b) O 1s and (c) N 1s for the SiO_2_–NH-SB and the corresponding Fe_2_O_3_ NP catalysts prepared via calcination (C) and chemical reduction (R).

XPS analysis of the NP catalysts was carried out to determine the chemical states of the iron oxide NP and assess the interaction of the ligand and the NPs. Figure 4a shows the Fe 2p spectrum for the SiO_2_–NH_2_–SB NP prepared by calcination (C) and a reducing agent (R), which are very similar. The Fe 2p_3/2_ is located at a BE of 711 eV and in good agreement with that reported for Fe_2_O_3_.^41^ The Fe 2p_3/2_ also has a clear shake up satellite peak at 718 eV, a characteristic of Fe^3+^, further indicating the presence of Fe_2_O_3_. Figure 4 (b) shows the O 1s core level before Fe adsorption with a dominant peak at 532.6 eV associated with the SiO_2_. After NP formation, an additional peak at a BE of 530.2 eV appears in the O 1s for catalysts prepared by calcination and chemical reduction, which is in excellent agreement with the BE for Fe–O–Fe, consistent with the formation of NPs.^42^ The O 1s core level of the other NP catalysts, shown in the the Supporting Information, Figure S5, displays the same trend with the appearance of the Fe–O–Fe peak in catalyst be annealing and chemical reduction, which is attributed to NP formation. The Fe 3p shown in the Supporting Information (Figure S6) is fit to a single peak with a BE of 55 eV, again indicating the presence of Fe_2_O_3_. Figure 4c shows the N 1s core level of the SiO_2_–NH_2_–SB catalyst, with a dominant peak at a BE of 399 eV, due to the amine functional group and a shoulder peak at 401 eV, attributed to protonated amines, as aminosilane protonation occurs from the support Si–OH.^29^ The peak intensity of the N 1s in the calcined catalyst decreases substantially, as excepted, due to loss of the ligand, but there are residual ligands present. The Si/N ratio estimates that ∼85% of the ligands are removed. The N 1s peak of both the calcined and NaBH_4_-treated catalysts is upshifted to a BE of 339.8 eV, indicating charge transfer from the organic ligand to the Fe_2_O_3_ NPs. XPS analysis of the other catalysts also shows the same trend with the N 1s core level shifting upward (see the Supporting Information Figure S7).

### 2.2 Catalytic Evaluation in PET Glycolysis

**Figure 5:**
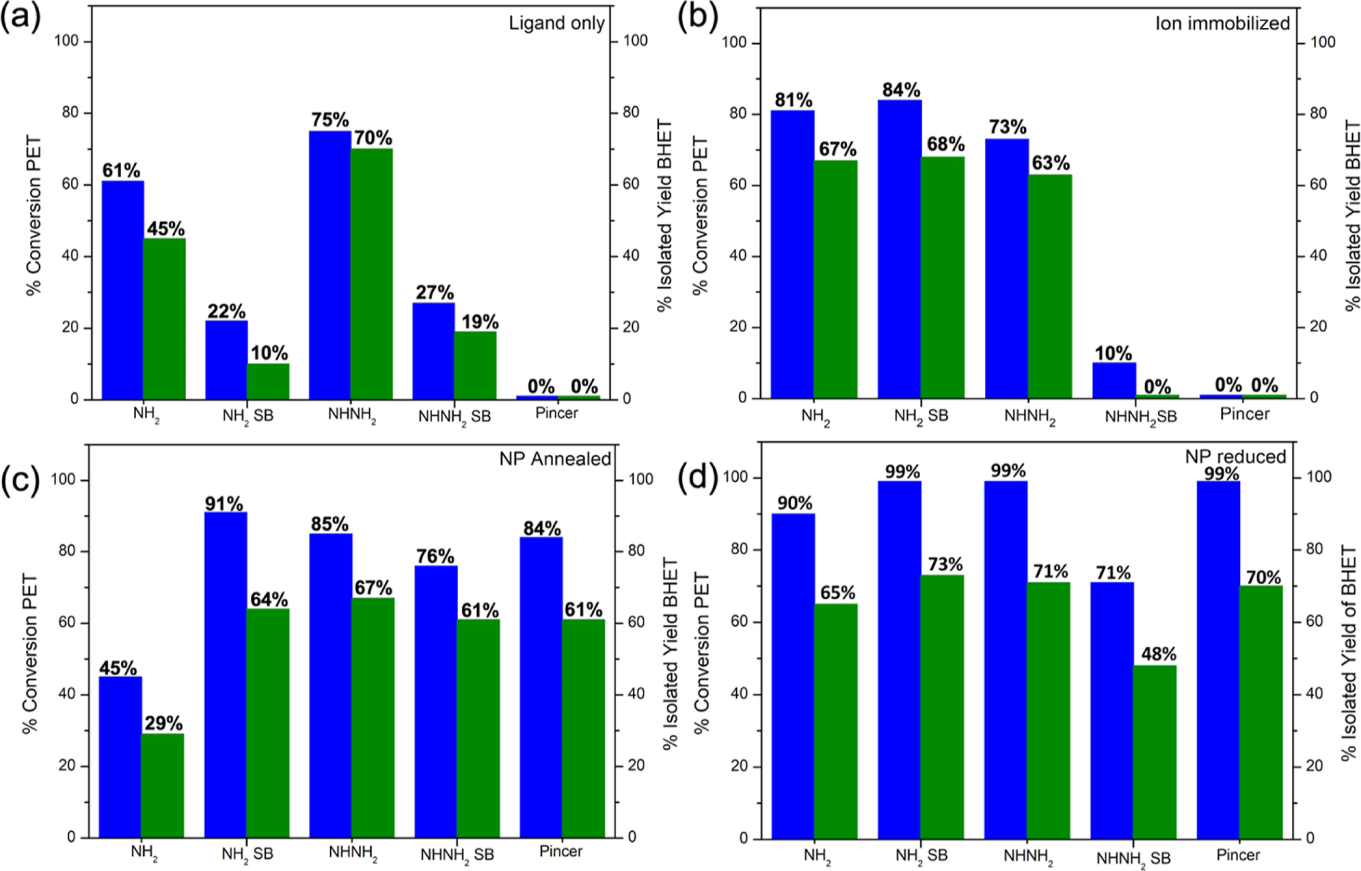
Percentage conversion of PET (blue) and isolated yield of BHET (green) obtained from glycolysis reaction catalyzed by (a) organic ligand modified SiO_2_ (before metal loading), (b) iron–ion-immobilized SiO_2_, (c) Fe_2_O_3_–NP SiO_2_ via calcination, and (d) Fe_2_O_3_–NP SiO_2_ prepared by chemical reduction.

The catalytic performance of all 20 catalysts for the glycolysis of PET was investigated under the same reaction conditions. Figure 5a–d compares the PET conversion and isolated yields of BHET for (a) SiO_2_ modified with the organic ligands, that is, before metal loading, (b) the Fe-ion-based catalysts (c), the Fe_2_O_3_ NPs prepared by calcination (d), and the ligand-Fe_2_O_3_ NP catalysts prepared by chemical reduction. It is worth noting that the analysis of the supernatant showed residual BHET remaining in the solution left after recrystallization, but no BHET dimer was detected by NMR. The activity of the functionalized SiO_2_ was tested as reference before the immobilizing the iron ion precursor. Interestingly, the SiO_2_ consisting only of the organic ligand attached to the surface displayed moderately good PET conversion as shown in Figure 4a. Good catalyst activity corresponded to organic ligands with terminal primary amines, that is, the NH_2_ and NH–NH_2_ ligands, having a conversion of 61 and 75%, respectively. This activity is associated with the ligands being organic bases and depolymerization occurred by based-catalyzed glycolysis of PET. Strong organic bases such as guanidines are popular for homogeneous catalyzed PET glycolysis and are attractive as they offer a metal-free depolymerization route but organocatalysts can lead to contamination in the monomer product. While we did not further investigate the catalytic activity of the organically functionalized, that is, metal-free SiO_2_ in this study, the results in Figure 4a nevertheless illustrate the potential of using heterogeneous organocatalysts for PET glycolysis. The bare SiO_2_ support did not catalyze the reaction to any extent.

Figure 5 compares the catalytic results after Fe-ion immobilization. The PET conversion increased for Fe immobilized on the SiO_2_–NH_2_, and SiO_2_–NH_2_–SB, with conversions of 81 and 84%, respectively, which is expected due to the presence of Fe^3+^ ions. A notable conclusion from Figure 5b is the considerable difference in activity observed between the Fe ions immobilized on SiO_2_ using the two Schiff base ligands. High conversion was observed for Fe-ion immobilized using the SiO_2_–NH_2_–SB ligand (84%), however only a 10% conversion was observed when using a SiO_2_–NH–NH_2_–SB ligand. While the Fe loading used in the reaction was equivalent, the organic ligand coverage on the SiO_2_–NH_2_–SB catalyst (74% surface coverage) is much greater than that of the SiO_2_–NH–NH_2_–SB (37% surface coverage) as previously described by the solid-state NMR analysis, indicating that the organic ligand and coordination chemistry of the iron on the surface-modified SiO_2_ plays a crucial role in the observed catalytic behavior. The Fe^3+^ ions immobilized using the pincer ligand did not catalyze the reaction to any extent, which was attributed to the tight binding of the Fe ions to the ligand.

Figure 5c,d shows the PET conversion and isolated yields obtained by using the NP catalysts obtained by calcination and chemical reduction, respectively. Generally, the NP catalysts displayed similar or higher PET conversions and BHET yields compared to their ion-immobilized version, regardless of whether they were prepared by calcination or reduction, with exception of one catalyst: SiO_2_–NH_2_. The conversion of PET decreased from 81% in the ion SiO_2_–Fe^3+^–NH_2_ catalyst to 45% when the catalyst was converted to NPs by calcination. However, when the catalyst was prepared by a chemical reduction, a conversion of 90% was obtained. This trend is attributed to the dual catalytic depolymerization, as reported by Dove and coworkers,^43^ due to the preservation of the amine ligands, which are catalytically active in the reaction, as also seen in Figure 5a. All the NP catalysts prepared by chemical reduction were superior to catalysts prepared by calcination, attributed to the formation of smaller NPs, and also the presence of the amine ligands that remain on the surface. In addition to the based catalysis glycolysis, synergistic effects have been shown to occur between metal salts and organic additives for PET glycolysis under homogeneous conditions. A recent report showed the addition of anisole to homogeneous PET glycolysis using alkali metal salts facilitated a lower reaction temperature.^44^ DFT calculations suggested that the electron-donating methoxy group in anisole transfers electron density to the carbonyl O atom making it more nucleophilic and thereby susceptible to attack by metal-ion species. The electron-donating ability of N containing groups in the ligands, as evidenced by XPS analysis, may have a similar effect in aiding glycolysis. Additionally, the role of H-bonding using N-containing organocatalysts for PET glycolysis has been shown to be important in activating the carbonyl group of PET and the −OH of EG.^45^

The catalytic evaluation highlights some important features of catalyst design for PET glycolysis: (i) the organic ligand used to bind the metal-ion can significantly impact the catalytic performance as illustrated by the different reactivity behavior, (ii) the metal oxide NP catalysts are in general superior to metal-ion-immobilized catalysts, and (iii) the presence of both the organic ligand and metal oxide NP (i.e., catalysts prepared by chemical reducing agent) gave the best performance. We used DFT calculations to rationalize the observed catalytic trends and gain further insights into the reaction.

First, we analyze the strength of the interaction between the Fe^3+^ metal cation and the functionalized surface, by calculating the interaction energies (Δ*E*_int_, see the Experimental Section in the Supporting Information for details). We performed optimizations of the functionalized SiO_2_/Fe^3+^ surfaces considering three possible electronic spin states, translated to three different electronic spin multiplicities (*M* = 2, 4, 6), showing that the high-spin *M* = 6 is the ground state (see the Supporting Information, Table S3). The nature of the surface under study allows the SiO_2_ surface to be functionalized with up to three organic linkers in the same unit cell. The calculated Δ*E*_int_ values represented in Table 2 are for the different organic linkers containing one, two, or three ligands (1L, 2L, and 3L, respectively).

**Table 2:** Calculated Interaction Energies (Δ*E*_int_, in kcal·mol^–1^) for the Fe^+3^ Ion Coordination with the Organic Ligands Supported on the SiO_2_ Surfaces in the Solvent Phase (Water)

structure	1L	2L	3L
SiO_2_–NH_2_	–1.66	–80.73	–143.30
SiO_2_–NH_2_–SB	–40.90	–172.47	–200.58
SiO_2_–NH–NH_2_	–123.83	–173.80	–258.27
SiO_2_–NH–NH_2_–SB	–84.48	–227.11	–303.82
SiO_2_–pincer	–132.54	–273.49	–290.01

**Figure 6:**
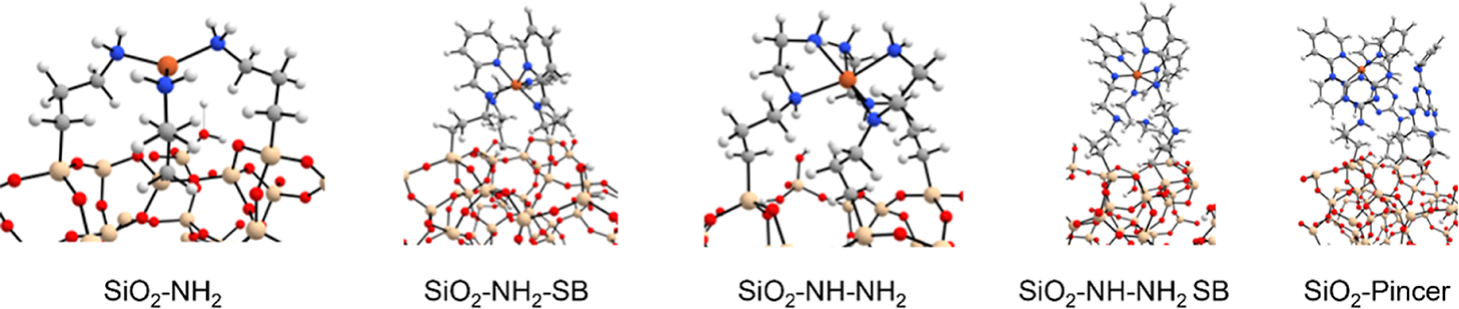
Optimized geometries for the different SiO_2_/ligands coordinating the Fe^3+^ ion. Color scheme: gray (C), white (H), blue (N), orange (Fe), red (O), and beige (Si).

The organic ligands present a similar trend with Δ*E*_int_ dramatically decreasing as fewer ligands are involved, meaning that the iron ion is trapped within a net of coordinating bonds between the organic ligands (see Figure 6, where the SiO_2_-ligand structures interacting with Fe^3+^ are shown). The two poorest performing catalysts, Fe^3+^ immobilized on SiO_2_–NH–NH_2_–SB and SiO_2_-pincer, have the most favorable Δ*E*_int_ values as shown in Table 2, which effectively traps the ion in between the three sterically bulky organic ligands. For the SiO_2_-pincer catalyst, Fe^3+^ is coordinated with six N groups of the three pyridine rings. For the SiO_2_–NH–NH_2_–SB catalyst, the Fe^3+^ is coordinated to the three imine and the three pyridinic N groups. For these ligands, Fe^3+^ presents the most favorable interaction energies, the metal being coordinated in an octahedral geometry and so sterically blocked from reacting, giving poor performance as observed. In the SiO_2_–NH_2_–SB catalyst Fe^3+^ is coordinated between three pyridinic Ns and two imine groups but, unlike the SiO_2_–NH–NH_2_–SB catalyst, the metal-ion is not octahedrally coordinated, resulting in a slightly lower Δ*E*_int_ value. This less Δ*E*_int_ and lower coordination geometry results in a less sterically hindered Fe^3+^ center, which would contribute to a better catalytic performance of the SiO_2_–NH_2_–SB catalyst (84% conversion) compared to the SiO_2_–NH–NH_2_–SB catalyst (10% conversion). A highly favorable metal-ion Δ*E*_int_ is also attributed to why the performance for the SiO_2_–NH–NH_2_ catalyst did not improve after Fe-ion immobilization. In this system, the ligand only catalyst demonstrated relatively good reactivity with 75% PET conversion. After Fe-ion immobilization (loading 4043.3 ppm), there was no change in the PET conversion (73%). In this catalyst, the Fe^3+^-ion is coordinated to six amine groups, combining three primary amines and three secondary amines, in an octahedral geometry. The high interaction energy limits the participation of the Fe^3+^ in the reaction and furthermore, the presence of Fe^3+^ also prevents the terminal amine groups from catalyzing the reaction. This is in contrast with the SiO_2_–NH_2_ catalyst where Fe^3+^ is coordinated only by three amine groups giving it a lower BE and so the PET conversion increased after Fe^3+^ immobilization.

The glycolysis reaction using the Fe^3+^ion and the NP catalysts were also modeled. The glycolysis of PET is the molecular degradation of the polymer by ethylene glycol (EG) through a transesterification reaction, where the ester linkage breaks and is transformed into hydroxyl groups (see the Supporting Information, Figure S7). The reaction process starts with the coordination of PET to the metal center by the oxygen of the carbonyl group of the ester. Since both ester groups are separated by only two –CH_2_ it is possible to face the coordination of both adjacent ester groups. The following step is the transesterification of the ester by the EG, where the carbonyl carbon is attacked by the free electron pair present on the hydroxyl group of EG. This is followed by the binding between the hydroxyl ethyl group of EG with the carbonyl carbon of PET, resulting in breaking the long polymer chain into two short oligomers. The subsequent glycolysis will break this oligomer forming the BHET product.

**Figure 7:**
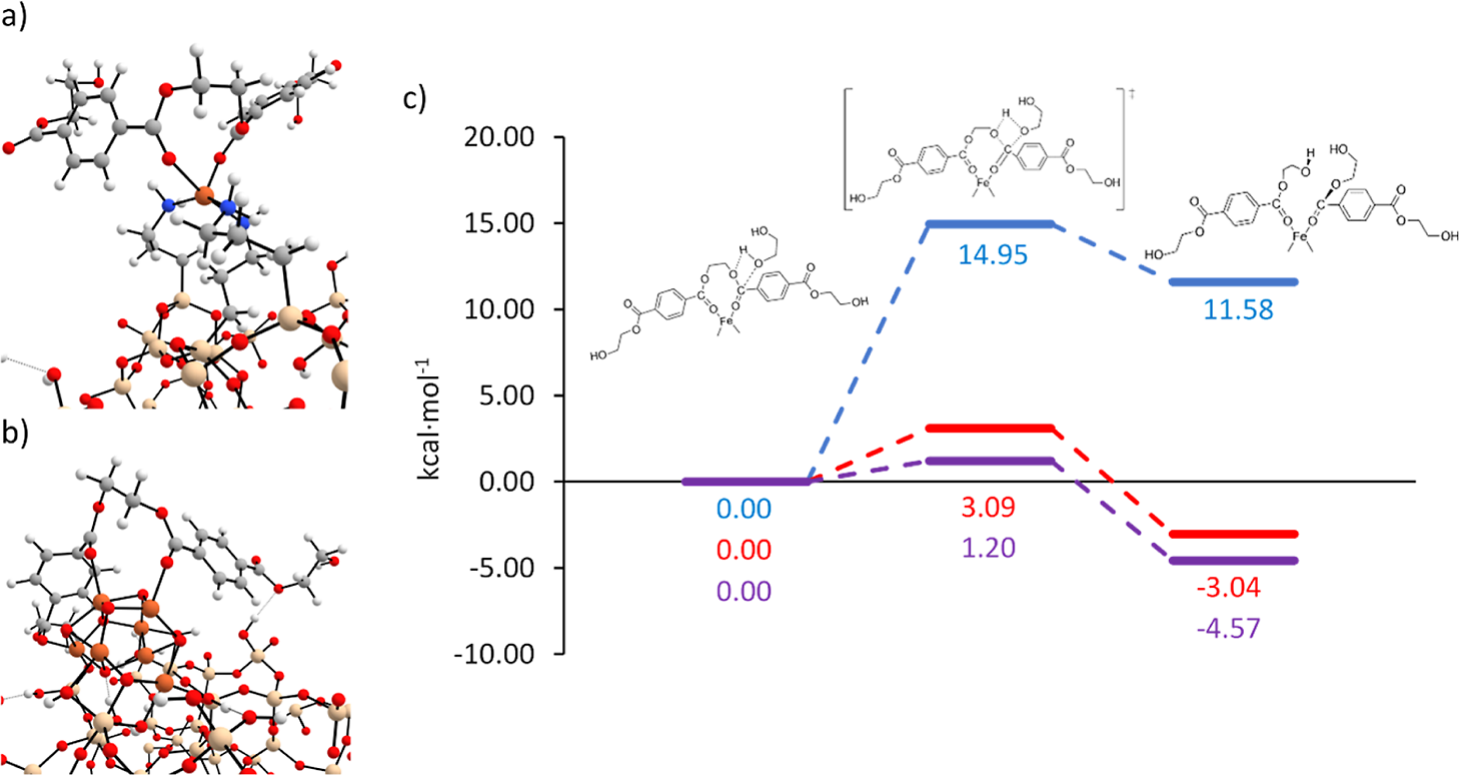
(a) Optimized geometry of the adsorbed PET molecular cluster over the Fe^3+^ ion. (b) Optimized geometry of the adsorbed PET over the iron oxide NC. Color scheme: gray (C), white (H), blue (N), orange (Fe), red (O, and beige (Si). (c) Relative Gibbs energy profiles at 190 °C (in kcal·mol^–1^) for PET glycolysis considering EG as a solvent, via uncatalyzed (blue color), catalyzed by a single atom Fe^3+^ (red color), and catalyzed by the iron oxide nanocluster (purple color).

This mechanism for PET depolymerization was modeled here in the absence and in the presence of either a single Fe^3+^-ion-immobilized catalyst or the NP catalyst (see Figure 7). In these simulations, two repetitive units of the PET polymer (A) were accounted for. In modeling the Fe^3+^-ion-immobilized catalyst, it is important to consider the availability of the metal center as active site. Therefore, systems where the Fe^3+^ ion is fully coordinated or trapped between the most voluminous ligands will not perform well due to steric hindrance (e.g., SiO_2_–NHNH_2_–SB and SiO_2_–pincer), as experimentally demonstrated. Therefore, the system selected to represent the single Fe^3+^-ion catalysts was the SiO_2_–NH_2_ catalyst (see Figure 7a). Although this is the system presenting the less favorable Δ*E*_int_, it is the structure presenting the least steric impediments. The NP catalyst, to keep the simulations computationally affordable, has been modeled here as an iron oxide nanocluster using the bulk cut nanoparticle model (BCN-M), a computational tool that automatically generates Wulff-like nanoparticle and nanocluster models for binary materials with controlled stoichiometry.^46^ This program, by introducing all the data relative to the Fe_2_O_3_ surfaces, generated as a minimal cluster a species of stoichiometry Fe_6_O_8_, in which four iron centers have a formal oxidation state of Fe^3+^ and the other two Fe^2+^. This composition makes our NC model have 28 total of unpaired electrons (five unpaired electrons for the four Fe^3+^ cations and four unpaired electrons for the two Fe^2+^ cations), rendering our NC to have a total electronic spin multiplicity of 29. Although the nanocluster (NC) does not perfectly match an Fe_2_O_3_ system (due to technical limitations of the software), since the structural modeling of nanostructures of binary ionic systems is a highly delicate task, we assume that the BCN-*M*-generated Fe_6_O_8_ NC is reasonable enough to reproduce the chemistry of a Fe_2_O_3_ supported on a SiO_2_ surface. Thus, we simulated the PET decomposition catalyzed by Fe_2_O_3_ NPs, using the adsorbed Fe_6_O_8_ NC on a silica surface as a catalyst model (see Figure 7b).

Figure 7 depicts the potential energy surfaces (PESs) for the uncatalyzed reaction and those catalyzed by single Fe^3+^ immobilized on SiO_2_–NH_2_ and by the NC catalyst. For the catalyzed reaction, the inset figure shows the PET fragment coordinates with the metal center through the carbonyl group (Fe–O=C). The transesterification reaction mechanism proposed here goes through a four-membered ring transition state (TS_A-B_) where the carbonyl carbon of the PET molecular cluster is attacked by the hydroxyl group of the EG subsequently forming the hydroxyl–carbonyl bond. Energetic values show that the uncatalyzed reaction not only goes through a high energy barrier but also presents an endergonic character. The activation energy is significantly reduced when performing the reaction with the Fe^3+^ and NC catalysts, which are similar but lower in the latter case (3.09 kcal mol^–1^ vs 1.20 kcal mol^–1^, respectively). The reactions with Fe^3+^ and the NC also revert the thermodynamics, becoming exoergic processes. Interestingly, reaction free energies are slightly more favorable than those catalyzed by Fe^3+^, −4.57 and −3.04 kcal mol^–1^, respectively), as the NC provides more anchoring points that confer stability to the products. Finally, for the sake of comparison, we also performed the same study using a larger NC with stoichiometry (Fe_2_O_3_)(FeO)_11_, that is, with a predominance of formally Fe^2+^ species (see Table S4). Interestingly, the energy barriers are similar for the two NC types (1.35 kcal mol^–1^ on the larger NC) but differ on their reaction energies, i.e., that associated with the larger NC is dramatically more favorable (−16.20 kcal mol^–1^), which also due to the presence of more interacting points that stabilize the newly formed products. Therefore, when performing the glycolysis reaction catalyzed by the NP based catalysts, it is enhanced not only kinetically as the activation energy is lower but also, thermodynamically, favoring the products formation. The increased exergonic character is the main difference between the single Fe^3+^-ion catalyst and the NP system, due to stabilization of the products and so therefore the metal oxide NP catalyst show superior reaction performance.

**Figure 8:**
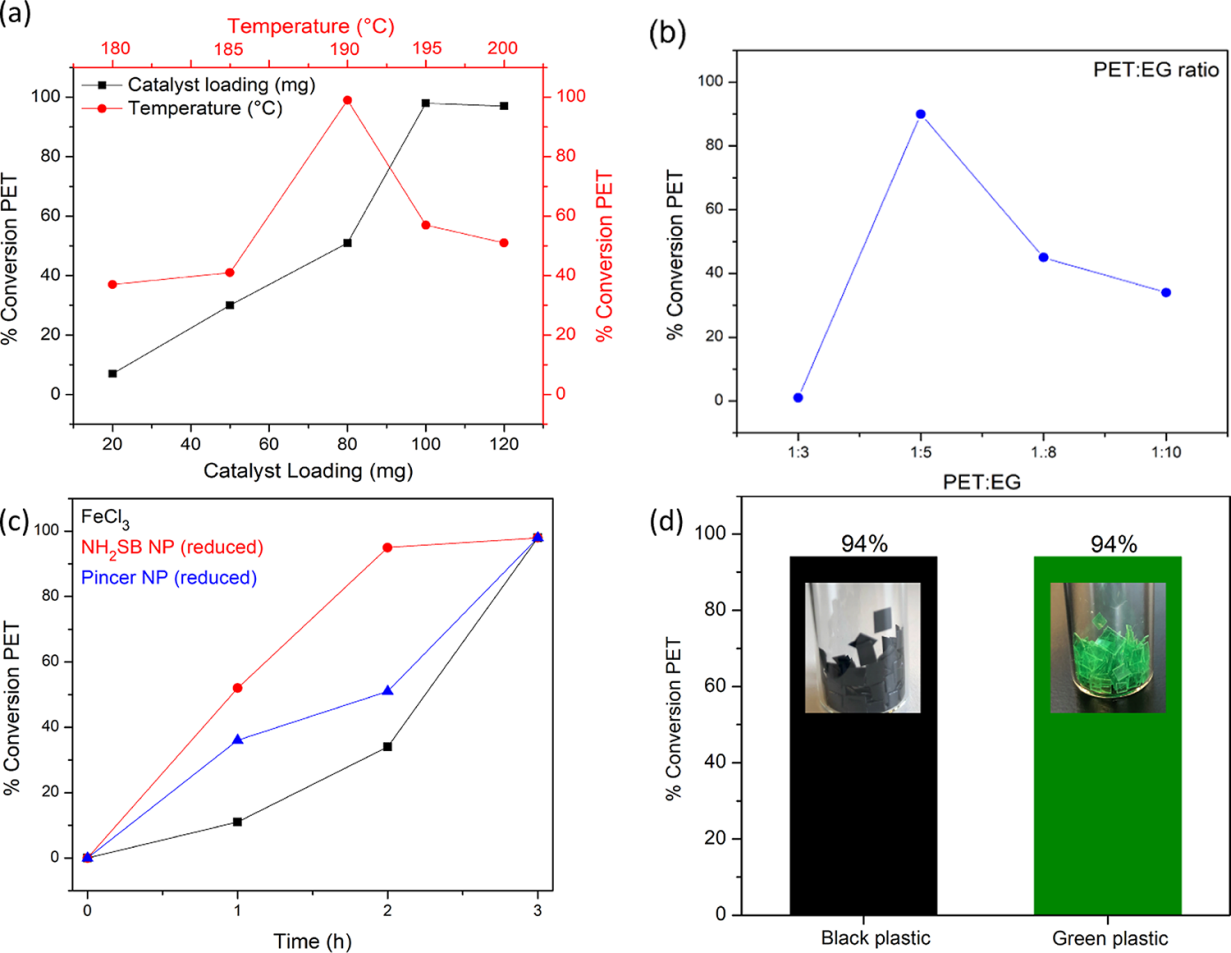
(a) Optimization of catalyst loading (w.r.t the SiO_2_ mass) and reaction temperature, (b) PET/EG ratio optimization, (c) time series of FeCl_3_, SiO_2_–Fe_2_O_3_ pincer NP and SiO_2_–Fe_2_O_3_–NH_2_SB NP both prepared via a reducing agent. (d) Application of heterogeneous catalysts to colored postconsumer PET.

Finally, we used the best performing catalyst as identified by the evaluation study, that is, the Fe_2_O_3_ NP catalysts prepared by the reduction using the SiO_2_–NH_2_–SB and SiO_2_–pincer ligands, to further study the impact of reaction parameters such as temperature, time, catalyst loading, and PET/EG ratio. Figure 8 compares the effect of reaction temperature and catalyst concentration. The PET conversions decreased at lower catalyst loading reaching full conversion at 100 mg of SiO_2_ catalyst (0.4 wt % Fe). The optimal temperature of the reaction was found to be 190 °C, with PET conversions decreasing at lower temperatures. Figure 8b shows the ratio of PET/EG was critical for the reaction with a ratio of 1:5 being optimal for full PET conversion. On decreasing the ratio to 1:3, there was insufficient EG to cover the PET for the reaction to proceed smoothly. Figure 8c shows a reaction time series, carried out under the same conditions and iron loading, ranging from one to three h for the heterogeneous SiO_2_ catalysts compared with a homogeneous FeCl_3_ catalyst. Interestingly, while all catalysts could achieve full PET conversion, both the heterogeneous catalysts display higher PET conversions compared to the homogeneous catalyst. It is clear that under these conditions, the Fe_2_O_3_ NP catalyst outperformed the ion-immobilized catalyst but also a homogeneous catalyst of equivalent Fe loading. This is attributed to the small diameter and high dispersion of the NPs on the SiO_2_ support, as evidenced by TEM and the presence of the organic ligands, which have a promotional effect on the PET glycolysis due to being organic amines. Figure S9 shows the recyclability of the SiO_2_–NP pincer catalyst tested over five cycles. The catalyst achieved full PET conversion on two reaction recycles. The conversion does drop after the third cycle (82%) but still displays favorable recyclability given the low loading metal (0.4 wt %) of the catalyst. An additional benefit of using a heterogeneous catalyst was the very low metal contamination in the BHET product, which was analyzed by ICP-MS (see the Supporting Information Table S2). Metal contamination of the BHET monomer from the catalyst in PET glycolysis often necessitates additional purification steps. BHET obtained using FeCl_3_ as the catalyst contained 4.9 ppm of Fe and visually had a pale yellow-orange color, while the BHET obtained using the heterogeneous catalyst was colorless, with an Fe concentration measured to be 1.3 ppm. Finally, the use of additives and colorants are commonly found in postconsumer plastic, and their impact on catalyst performance is not always explored. The Fe_2_O_3_ NP catalyst was used to depolymerize black and green colored PET as shown in Figure 8d, giving high PET conversions of 94% for both black and green plastic showing that this catalyst can effectively be used for colored postconsumer PET.

## 3 Conclusions

In conclusion, we investigated the catalyst design for the glycolysis of PET using heterogeneous mesoporous SiO_2_ catalysts. The SiO_2_ support was modified with a range of N containing organic ligands used for the immobilization of Fe ions. Solid-state NMR allowed for the investigation of the structures of the modified SiO_2_ supports, confirming the successful grafting of the ligands and revealing the distinct types of silicon environments. Surface coverages were found to be between 40 and 73% based on quantitative ^29^Si NMR experiments. The Fe-ion-immobilized catalysts were converted into Fe_2_O_3_ NP supported catalysts by calcination or treatment with aqueous NaBH_4_ as confirmed by TEM and XPS. In general, the NP-based catalysts performed better than their ion-immobilized catalysts and the presence of both the Fe_2_O_3_ NP and organic ligand displayed the best performance giving full PET conversion and highest BHET yields. The ligand-assisted Fe_2_O_3_ NP catalyst also outperformed a homogeneous FeCl_3_ catalyst. The superior performance of the ligand-stabilized Fe_2_O_3_ NP catalyst was attributed to cooperative effects between the organic ligand and the NPs. The N-containing amine ligands facilitated base-catalyzed glycolysis of PET, similar to homogeneous organocatalysts and also behaved as Lewis bases to facilitate electron transfer to the carbonyl group making it more susceptible to nucleophilic attack by the metal catalyst. This work shows the significant potential of nanoparticle-based heterogeneous catalysts for PET glycolysis and the use of organically functionalized support materials to further enhance catalytic activity.

## Supporting Information Available

Comparison of heterogeneous catalysts in the literature, catalyst synthesis and DFT computation details, SEM images of unfunctionalized, ion-immobilized SiO_2_ and NP-immobilized SiO_2_, Si 2p, Fe 3p, O 1s, and N 1s XPS of catalysts, NMR of BHET product, ^13^C solid-state NMR spectra of functionalized SiO_2_, ICP-MS analysis for Fe quantification, and calculated relative Gibbs energies for the modeled reaction systems, catalyst recyclability evaluation (PDF)
