# Supplementary material for: Ligand-Aided Glycolysis of PET Using Functionalized Silica-Supported Fe2O3 Nanoparticles
Source: ACS Sustain Chem Eng. 2023 Oct 18;11(43):15544–55. doi: 10.1021/acssuschemeng.3c03585 (PMC10618922; doi:10.1021/acssuschemeng.3c03585)
Supplement: Supplementary file 1 — sc3c03585_si_001.pdf [file sc3c03585_si_001.pdf]

Supporting Information

Ligand-Aided Glycolysis of PET using Functionalized Silica Supported Fe_2O_3 Nanoparticles

Éadaoin Casey[1,2], Rachel Breen[1,2], Jennifer S. Gómez[3], Arno P. M. Kentgens[3], Gerard Pareras[4], Albert Rimola[4], Justin. D. Holmes[1,2], Gillian Collins[1,2]*

[1] School of Chemistry, University College Cork, Cork, T12 YN60, Ireland.

[2] AMBER Centre, Environmental Research Institute, University College Cork, Cork, T23 XE10, Ireland.

[3] Institute for Molecules and Materials, Radboud University, Nijmegen, 6525 AJ ,The Netherlands.

[4]Departament de Química, Universitat Autònoma de Barcelona, 08193 Bellaterra, Catalonia, Spain.

*To whom correspondence should be addressed: Tel: +353 (0)21 4205143. E-mail: g.collins@ucc.ie

Supporting Information Contents

Item	Title	Page no.
Table S1	Heterogeneous catalyst reported in the literature	S3
Figure S1	^1H liquid NMR spectrum of the BHET product formed from the glycolysis of PET.	S10
Figure S2	XPS analysis of the Si 2p core level for bare SiO_2 and the functionalized SiO_2	S11
Figure S3	$^1H \rightarrow ^{13}C$ CPMAS solid state NMR spectra of Schiff base ligand attached to (a) SiO_2-NH_2 (Ligand 2) and (b) SiO_2 – $NHNH_2$ ((Ligand 4) catalyst.	S12
Table S2	ICP-MS for Fe loading for each ion and NP catalyst	S13
Figure S4	SEM images of (a) mesoporous cellular foam (b) ion-immobilized SiO2 (c) NP immobilized SiO_2.	S13
Figure S5	Fe 3p XPS spectrum of SiO_2-pincer catalyst indicating the presence of Fe_2O_3.	S14
Figure S6	O 1s core level XPS spectra of ligand only catalyst sand NP catalyst prepared by calcination (C) and using $NaBH_4$ (R) for SiO_2-NH_2, SiO_2-$NHNH_2$, SiO_2-$NHNH_2$ SB and SiO_2-pincer ligands.	S14
Figure S7	N 1s XPS core level spectra of ligand only and both calcined and chemically reduced NP catalysts for SiO_2-NH_2, SiO_2-$NHNH_2$, SiO_2-$NHNH_2$ SB and SiO_2-pincer ligands.	S15
Table S3	Calculated relative energies (in kcal·mol^{-1}) for the three different spin multiplicities, doublet (D), quadruplet (Q) and sextet (S), for the Fe^{+3} ion using the SiO_2-NH_2 system as a test case.	S15
Figure S8	Mechanism for the PET glycolysis reaction.	S15
Table S4	Calculated relative Gibbs energies for the modelled reaction systems, considering 463.15 K and ethylene glycol (EG) as a solvent ($\Delta G_{EG(463.15\ K)}$), including the uncatalyzed process, and the reaction catalyzed by the Fe^{+3} ion, the Fe_2O_3 nanoparticle ($(Fe_2O_3)_2(FeO)_2$) and the FeO nanoparticle ($(Fe_2O_3)(FeO)_{11}$).	S16

Table S1: Summary of literature reports using heterogeneous catalysts for PET glycolysis

Entry	Catalyst	Catalyst Loading (wt %)	Temp (°C)	Time (min)	BHET Yield	Ref
1	Review					1
2	n-ZnO	5%	190	60	90	2
3	ZnO	1%	260	60	67	3
4	Co_3O_4	2%	260	60	63	3
5	Mn_3O_4	2%	260	60	74	3
6	Co NPs	1.5%	180	180	77	4
7	ZnO	1%	196	120	50	5
8	CoO	1%	196	120	10	5
9	CoZnO	1%	196	120	80	5
10	ɣ-Fe_2O_3	1%	300	60	90	6
11	CeO_2	1%	300	60	25	6
12	Fe^{III} nanosheet	1%	200	30	100*	7
13	Fe_2O_3-MWCNT	5%	190	120	100*	8
14	MWCNT	5%	190	120	78*	8
15	Magnetite	5%	190	120	62	8
16	Titanate nanotubes	0.3%	196	120	84	9
17	Na- Titanate nanotubes	0.3%	196	180	80	10
18	Zn- Titanate nanotubes	0.3%	196	180	87	10
19	GO-Mn_3O_4	-	300	80	97*	11
20	MnO_2/HGO	0.01%	200	10	100*	7
21	ɣFe_2O.NGO	9%	195	180	100*	12
22	Pd/h-BN	0.01%	100	30	92*	13
23	Fe_2O_3NPs@h-BNNs	0.01%	200	300	100*	13
24	Fe_3O_4@SiO_2@(mim)[$FeCl_4$]	15%	180	1440	100*	14
25	rGO\[TESPMI]$_2$$CoCl_4$	15%	190	180	95	15
26	hydrocalcites;Mg:Al molar ratio 2	1%	196	50	61	16
27	hydrocalcites; Mg:Al molar ratio 3	1%	196	50	66	16
28	hydrocalcites;Mg:Al molar ratio -4	1%	196	50	31	16
29	Calcined hydrocalcites; Mg:Al molar ratio 2	1%	196	50	76	16
30	Calcined hydrocalcites; Mg:Al molar ratio 3	1%	196	50	81	16
31	Calcined hydrocalcites; Mg:Al molar ratio 4	1%	196	50	66	16
32	Mg-Al-O@Fe_3O_4	0.1%	240	90	80	17
33	ZnAl-1	1%	196	85	66	18
34	ZnAl-2	1%	196	85	75	18
35	ZnAl-3	1%	196	85	76	18
36	ZnAl-4	1%	196	85	76	18
37	(Mg-Zn)-Al LDH	1%	190	180	75	19
38	S/Zn-Ti-300°C	0.3%	180	180	72	20
39	SCZ	0.3%	180	180	72	21
39	$ZnMn_2O_4$	1%	260	60	92	3
40	$CoMn_2O_4$	1%	260	60	89	3

41	$ZnCo_2O_4$	1%	260	60	81	3
42	$CoFe_2O_4$/C10-OAC	0.02%	195	150	95	22

* indicates the reported BHET was not an isolated yield.

1. Experimental

1.1 Chemicals and Materials

3-aminopropylytrimethoxysilane (APTMS), 3-(2-aminoethyl-aminopropyl)trimethoxysilane (AEAPTMS), 2-pyridine carboxaldehyde, cyanuric chloride (CNC), N,N diisopropylethylamine (DIEA), sodium borohydride ($NaBH_4$), iron (III) chloride ($FeCl_3.4H_2O$), ammonium hydroxide solution (30-33%), methanol, ethanol, toluene, acetonitrile, tetrahydrofuran and ethylene glycol were all purchased from Sigma Aldrich. Mesoporous cellular foam (MCF) SiO_2 was purchased from Glantreo Ltd. PET was obtained from wastewater bottles after labels and lids being removed were cut into 1 mm x 1 mm pieces for glycolysis experiments. Repeating unit of 192.68 g mol^{-1} was used as the molecular weight of PET.

1.2. Synthesis of surface modified mesoporous cellular foam SiO_2

Synthesis of SiO_2-NH_2 (Ligand 1): 2 g of MCF SiO_2 was placed in a round bottom flask along with 4 mL of 3-ATPMS and 75 mL toluene. The mixture was refluxed at 120 °C for 12 h under argon. Once reaction had completed, the solid product (SiO_2-NH_2) was suction filtered and washed with ethanol before dried at 60 °C.

Synthesis of SiO_2-NH_2-SB (Ligand 2): 1 g of the SiO_2-NH_2 prepared in the previous step, was placed in a round bottom flask along with 1.2 mL 2-pyridinecarboxaldehyde and 50 mL ethanol. The reaction mixture was refluxed at 60 °C for 24 h. Once completed, the solid product (SiO_2-NH-SB) was filtered and washed with ethanol before being dried at 60 °C.

Synthesis of SiO_2-$NHNH_2$ (Ligand 3): 2 g of MCF SiO_2 was placed in a round bottom flask along with 4 mL of AEAPTMS and 75 mL toluene. Mixture was refluxed at 120 °C for 12 h under argon. Once reaction

had completed, the solid product (SiO2-$NHNH_2$) was vacuum filtered and washed with ethanol before dried at 60 °C.

Synthesis of SiO_2-$NHNH_2$ SB (Ligand 4): 1 g SiO_2-$NHNH_2$ prepared in the previous step, was placed in a round bottom flask along with 1.2 mL 2-pyridinecarboxaldehyde and 50 mL ethanol. The reaction mixture was refluxed at 60 °C for 24 h. Once completed, solid product (SiO_2-$NHNH_2$ SB) was filtered and washed with ethanol before being dried at 60 °C.

Synthesis of SiO_2-Pincer (Ligand 5): 1.4 g SiO_2-NH_2, 1.8 g cyanuric chloride (CNC), 1.8 mL DIEA and 40 mL THF were added to a round button flask. The reaction mixture was left to stir in an ice bath under argon for 8 h. The product SiO_2-CNC was filtered off and washed with THF (5 x 20 mL) and dried overnight. In a three-neck round bottom flask, 1.5 g of the SiO_2-CNC was dispersed in 30 mL dry acetonitrile, 2 g 2-aminopyridine and 2 mL DIEA were added and refluxed for 48 h. After completion, the solid product (SiO_2-Pincer) was filtered off and washed with methanol (5 x 30 mL) and dried at 50 °C overnight.

1.3 Fe ion immobilization onto ligand modified SiO_2

Fe species were immobilized onto each of the functionalized SiO_2 supports by stirring 2 g $FeCl_3.4H_2O$ and 1 g of the ligand modified SiO_2 with 50 mL H_2O for 1 h. Once completed, 1 mL ammonium hydroxide solution was added, and pH of the solution adjusted from 2 to 12. The product was filtered off and washed with copious amounts of H_2O.

1.4 Synthesis of Fe_2O_3 NPs on SiO_2

The Fe ion-based catalysts were converted into iron oxide nanoparticle catalysts by calcination or chemical reduction. Catalysts were annealed in a tube furnace at 450 °C for 3 h in air. For chemical reduction method, 0.5 g of the Fe-ion immobilized SiO_2 were placed in a 50 mL beaker. A freshly prepared solution of $NaBH_4$ (0.095 g, 2.5 mmol) and 2 mL H_2O was rapidly added under vigorous stirring, turning the catalyst from orange to brown-black. The solution was stirred for 1 h before filtering and washing with copious amounts of H_2O.

1.5 Glycolysis reaction of PET to form BHET

In a typical procedure, 1 g of PET, 0.1 g catalyst and 5 mL of ethylene glycol were refluxed at 190 °C for 3 h. Once reaction was complete, 5 mL of ice-cold H_2O was added to the reaction filtered off and dried. The catalyst was separated by filtration. The filtrate was placed in the fridge overnight after which the BHET crystallized out the solution. The BHET crystals were collected be filtration, washed with water and dried overnight before being weighed. The percentage PET conversion and isolated BHET yield calculated be the equation 1 and equation 2, respectively.

$$\text{\% Conversion of PET: } \frac{W_0 - W_1}{W_0} \cdot 100 \qquad \textbf{Eqn. 1}$$

Where W_0 is the initial weight of the PET used and W_1 is the weight of the unreacted PET after the reaction.

$$\text{\% Yield of BHET: } \frac{W_{BHET}/M_{BHET}}{W_0/M_{PET}} \cdot 100 \qquad \textbf{Eqn. 2}$$

Where, W_{BHET} is the weight of the BHET product after recrystallization, M_{BHET} is the molecular weight of BHET, W_0 is the initial weight of PET used and M_{PET} is the molecular weight of the repeat unit of PET.

NMR analysis confirmed that no formation of dimers or oligomers occurred and that BHET was the only product formed in the reaction. The ^1H liquid-state NMR spectrum of BHET can be seen in the supplementary information Figure S1, where the four main peaks were a singlet at 8.12 ppm corresponding to the aromatic ring protons, an OH triplet at 5.01 ppm, a CH_2 triplet at 4.32 ppm and a quartet at 3.77 ppm for the CH_2 next to the OH group. Residual solvent d_6-DMSO was located at 2.51 ppm. The results are in good agreement with literature values of BHET.[23]

2. Materials Characterization

X-ray Photoelectron Spectroscopy (XPS) was acquired using a KRATOS AXIS 165 monochromatized X-ray photoelectron spectrometer equipped with an Al Kα (hv = 1486.6 eV) X-ray source. Spectra were collected at a take-off angle of 90° and all spectra were referenced to the C 1s peak at 284.8 eV.

Transmission electron microscopy (TEM) analysis was performed using a FEI Titan TEM, at an operating voltage of 300 kV. The iron loading in the catalyst was determined by inductively coupled plasma mass spectroscopy (ICP-MS) using an Agilent 7700 ICP-MS equipped with nickel-tip with copper base sampler cones. Autosamples (Agilent Technology, Japan) were used for all measurements. All measurements were performed in three replicates from each vial. In brief, the ICP-MS was operated in full quantitative mode with a Ni sampler and skimmer cones, MicroMist glass concentric nebulizer and quartz Scott-type spray chamber. Samples were quantified based on external calibrations constructed using standard solutions prepared on the day of analysis. Standard solutions were prepared from TraceCERT®, 1 mg/L Fe in nitric acid (Sigma-Aldrich, UK). Method blanks and certified references materials (Environmental Spike Mix, Agilent) were used to monitor the system performance and instrumental drift. Full data were recorder and analyzed with Agilent Mass Hunter Data Software (version 4.6 C.01.08).

^{1}H liquid-state NMR spectra were acquired on a Bruker Avance III NMR spectrometer operating at B_0 = 7.05 T (ν_0 = 300.13 MHz for ^{1}H), in proton-coupled mode. Samples were dissolved in deuterated chloroform ($CDCl_3$) or deuterated d_6-DMSO and tetramethysilane (TMS) was used as the internal standard.

Solid-state NMR experiments were carried out on numerous instruments. ^{29}Si NMR experiments were recorded at B_0 = 7.05 T (ν_0 = 300.13 MHz for ^{1}H) using a Varian VNMRS spectrometer, equipped with a 9.5 mm HX probes employing a MAS rate ν_R = 4 kHz. ^{29}Si one pulse excitation (SPE) experiments were acquired averaging 128 transients with a recycle delay of 1800 s, employing an rf-field strength of $\nu_1 \approx$ 42 kHz. The integrals for the Q^4 sites were corrected for the extremely T_1 extracted from saturation-recovery curves. $^{1}H \rightarrow ^{29}Si$ CPMAS CPMG experiments were recorded with a CP contact time of 2 ms, a recycle delay of 5 s, and 512 transients. For the CPMG train, each full echo was 6 ms in duration and a total of 12 echoes were collected. ^{29}Si chemical shifts were referenced using a solid sample of zeolite 4A (δ_{iso} = –89.70 ppm).

$^1H \rightarrow ^{13}C$ CPMAS NMR measurements for **SiO_2-NH_2 (Ligand 1)** and **SiO_2-NH_2-SB (Ligand 2)** were carried out on a Bruker Avance NEO 600 MHz spectrometer (B_0= 14.1 T) employing a Varian 3.2 mm HXY probe at ν_R= 14 kHz. The initial $\pi/2$ pulse was 2.17 µs corresponding to an rf-field of ~ 115 KHz and was followed by a CP contact time of 1 ms. The $^1H \rightarrow ^{13}C$ CPMAS spectra were recorded using recycle intervals of 8 s (Ligand 1) and 10 s (Ligand 2) and were the result of averaging 1024 transients. SPINAL ^1H decoupling[24] (~50 kHz) was applied during acquisition. The Hartmann-Hahn condition[25-26] was calibrated using a powdered sample of adamantane. ^{13}C chemical shifts were referenced using a solid sample of adamantane (δ_{iso} = 29.47 and 38.52 ppm).

$^1H \rightarrow ^{13}C$ CPMAS NMR measurements for SiO_2-$NHNH_2$ (Ligand 3), SiO_2-$NHNH_2$ SB (Ligand 4) and SiO_2-Pincer (Ligand 5) were recorded on a Bruker Avance NEO 850 MHz spectrometer (B_0= 19.97 T) using a Varian 3.2 mm HXY probe. The samples were spun at a MAS rate, ν_R = 15 kHz. The experiments were acquired under the following experimental conditions: ^1H 90° pulse was set to 2.55 µs corresponding to an rf-field of ~ 98 kHz. The VACP contact time was 1 ms (for ligands 3 and 4) and 10 ms (for ligand 5) using an 80-100 % ramp shape at the ^1H channel, while the RF nutation frequency on the ^{13}C channel was 65 kHz. The spectra were recorded using recycle intervals of 3.5 s (Ligand 3), 2.5 s (Ligand 4) and 7 s (Ligand 5) and were the result of averaging 2048 (Ligands 3 and 4) and 10240 (Ligand 5) transients. SPINAL ^1H decoupling[24] (~80 kHz) was applied during acquisition. The Hartmann-Hahn condition[25-26] was calibrated using a powdered sample of glycine. For Ligand 5; the $^1H \rightarrow ^{13}C$ CPMAS spectrum was acquired at –40°C to decrease the dynamics in the sample which reduced the CP efficiency. For all solid-state NMR experiments, rotors were packed in air. Data processing was carried out using Bruker TopSpin (version 4.1.3) and ssNake (version 1.3).[27]

The relative surface coverage obtained from the quantitative ^{29}Si MAS NMR spectra was estimated using equation 3 and expressed with respect to the bare SiO_2 support. The degree of condensation of the Si atoms was estimated using equation:[28]

$$100 - \left(\frac{(Q^2 + Q^3)ligand}{(Q^2 + Q^3)SiO_2} \cdot 100\right) \qquad \textbf{Eqn. 3}$$

$$\frac{\sum_{n=2}^{4} nI(Q^n)}{4} + \frac{\sum_{n=1}^{3} nI(T^n)}{3} \cdot 100 \qquad \textbf{Eqn. 4}$$

3. Density functional theory (DFT) calculations

Density functional theory (DFT) simulations were carried out using the CP2K package.[29] The mesoporous SiO_2 structure studied here has been computationally represented as a periodic amorphous silica (SiO_2) surface. The surface selected has a silanol density of 7.2 OH/nm^2 assuring the maximum number of Si atoms available to be functionalized in the unit cell.[30] For the initial SiO_2 surface, both internal atomic positions and cell parameters were optimized (resulting with dimensions of 13.34 x 13.73 x 49.21 Å^3. The following optimizations were performed only relaxing the geometry parameters. Optimizations were carried out using the semi-local Perdew–Burke–Ernzerhof (PBEsol) functional,[31] combined with a double-ζ basis set (DZVP-MOLOPT-SR-GTH Gaussian basis set) for all the atom types, together with the Grimme D3BJ correction term to the electronic energy,[32] and a cutoff set at 500 Ry for the plane wave auxiliary basis set. Core electrons were described with the Goedecker–Teter–Hutter pseudopotentials[33] and valence ones with a mixed Gaussian and plane-wave (GPW) approach.[34] Solvation effects were considered by performing a single point energy calculations on the optimized systems adopting the self-consistent continuum solvation (SCCS) model as implemented in CP2K, aiming to reproduce the experimental conditions of water and ethylene glycol as solvents.[35] Binding energies (BE) have been calculated by applying the counterpoise correction in order to avoid basis-set superimposition errors (BSSE). In CP2K, the interaction energy $\Delta \mathbf{E}_{\mathbf{int}}$ of the metal on the SiO_2 structure can be calculated defining 2 fragments **A** and **B**, as shown in equation 5.

$$\boldsymbol{E_{int}} = \boldsymbol{E_{AB}} - (\boldsymbol{E_A} + \boldsymbol{E_B}) \qquad \textbf{Eqn. 5}$$

where $\mathbf{E}_{\mathbf{AB}}$ is the absolute potential energy of the SiO_2/metal system, $\mathbf{E}_{\mathbf{A}}$ corresponds to the absolute potential energy of the bare silica and $\mathbf{E}_{\mathbf{B}}$ is the absolute potential energy of the naked metal.

Finally, to determine the nature of the stationary points of the potential energy surfaces (i.e., local minima and saddle points) the corresponding vibrational harmonic frequencies were calculated at the PBE-D3BJ/DZVP level using the finite differences method. A partial Hessian approach was used to reduce the computational cost of the calculations and the vibrational frequencies were calculated on the optimized geometries only for a fragment of the entire system, which included the metal centre and the organic ligands for the single atom Fe^{3+} structures and, the metal-nanocluster and the adsorbates for the FeO nanoparticle structures.

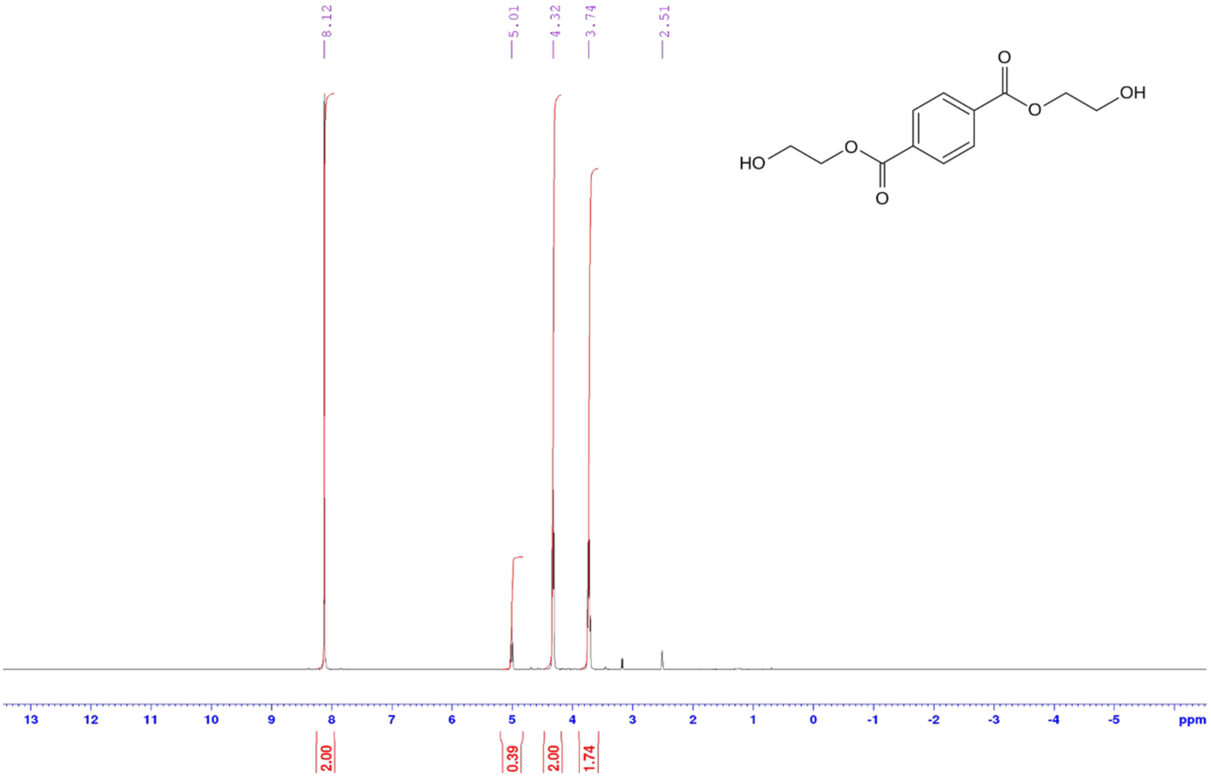

Figure S1: ^{1}H liquid NMR spectrum of the BHET product formed from the glycolysis of PET.

NMR analysis confirmed that no formation of dimers or oligomers occurred and that BHET was the only product formed in the reaction. The ^{1}H liquid NMR spectrum of BHET shows the four main peaks

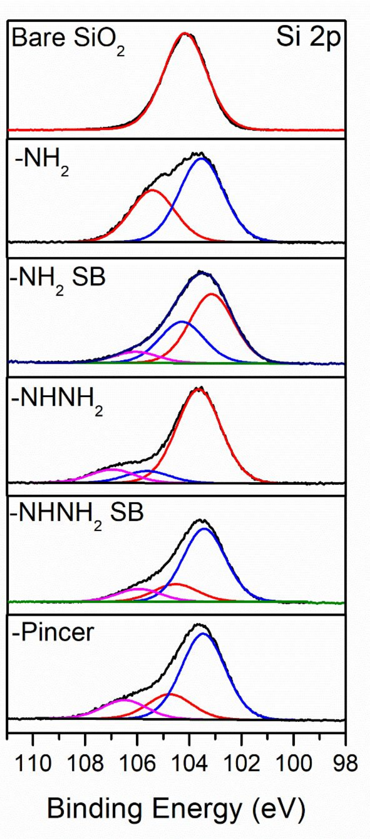

were a singlet at 8.12 ppm corresponding to an aromatic ring, an OH triplet at 5.01 ppm, CH_2 triplet at 4.32 ppm and CH_2 quartet at 3.77 ppm. Residual solvent d_6-DMSO was located at 2.51 ppm. The results are in good agreement with literature values of BHET.[1]

Figure S2: XPS analysis of the Si 2p core level for bare SiO_2 and the functionalized SiO_2 with -NH_2 (Ligand 1), -NH_2 SB (Ligand 2), -$NHNH_2$ (Ligand 3), -$NHNH_2$ SB (Ligand 4) and pincer (Ligand 5) ligands, respectively**.**

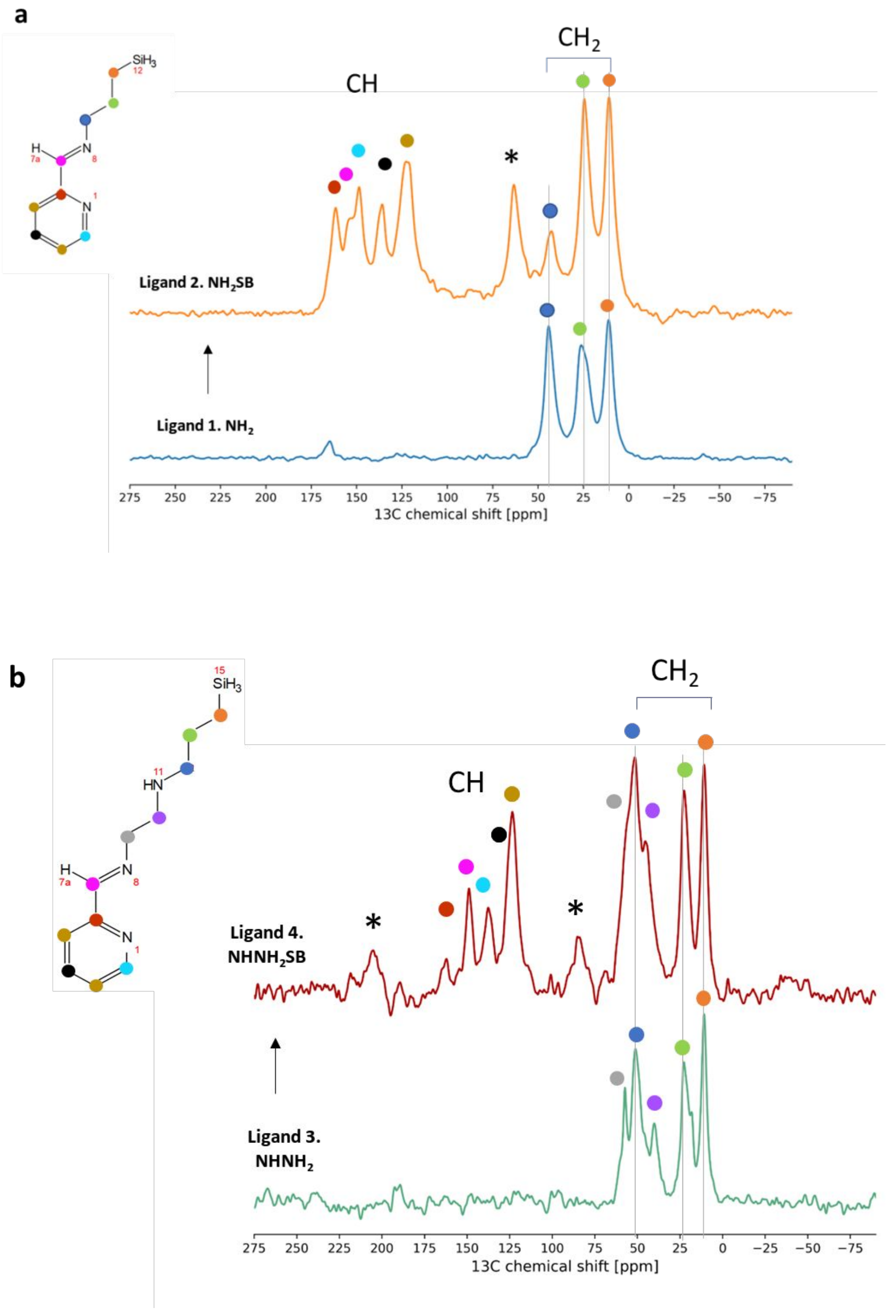

Figure S3: $^{1}H \rightarrow ^{13}C$ CPMAS solid state NMR spectra of Schiff base ligand attached to (a) SiO_2-NH_2 (Ligand 2) and (b) SiO_2 – $NHNH_2$ ((Ligand 4) catalyst. * Indicates the presence of additional peaks.

Table S2: ICP-MS for Fe loading for each ion and NP catalyst

Fe loading on SiO_2 supported catalysts	
**Catalyst**	**Fe Loading**
SiO_2-Fe-NH_2	4173 ppm
SiO_2-Fe NH_2SB	4981 ppm
SiO_2-Fe $NHNH_2$	4043 ppm
SiO_2-Fe $NHNH_2$ SB	3964 ppm
SiO_2-Fe pincer	3904 ppm
Fe metal contamination in BHET product obtained from different catalysts	
BHET obtained using $FeCl_3$ (homogeneous)	4.6 ppm
BHET obtained using SiO_2-Fe_2O_3 NP (heterogeneous)	1.3 ppm

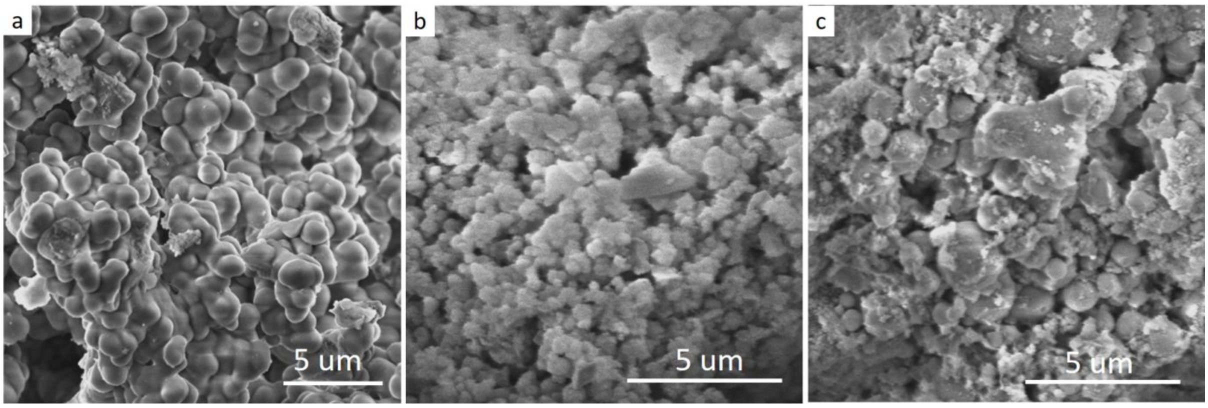

Figure S4: SEM images of (a) mesoporous cellular foam (a) Fe-ion-immobilized SiO_2 (c) Fe_2O_3 NP immobilized SiO_2.

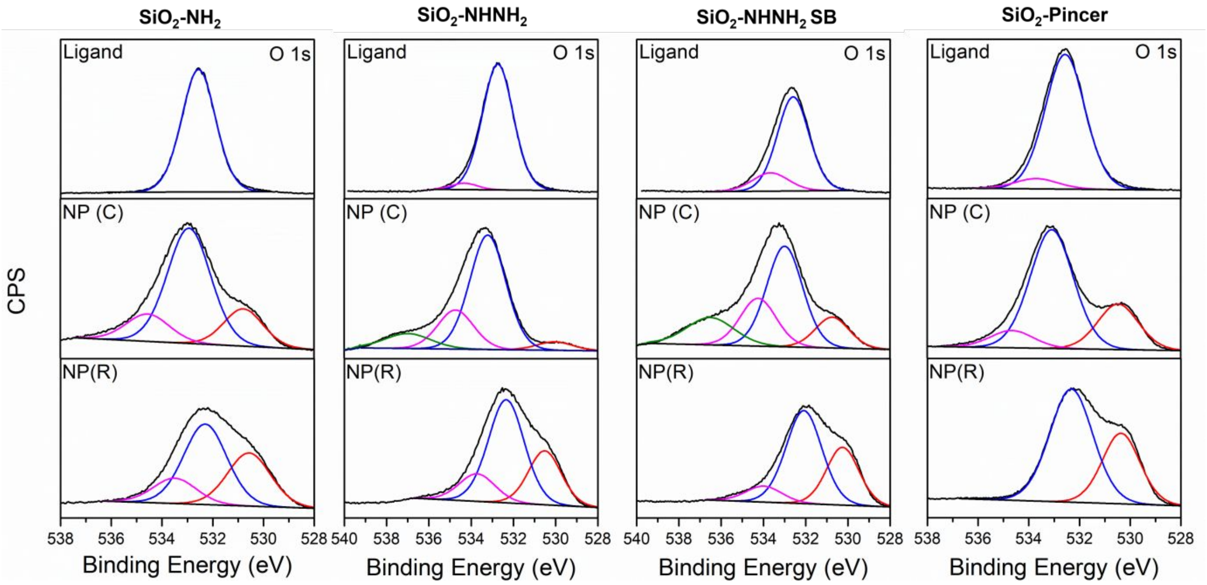

Figure S6: O 1s core level XPS spectra of ligand only catalyst sand NP catalyst prepared by calcination (C) and using $NaBH_4$ (R) for SiO_2-NH_2, SiO_2-$NHNH_2$, SiO_2-$NHNH_2$ SB and SiO_2-pincer ligands.

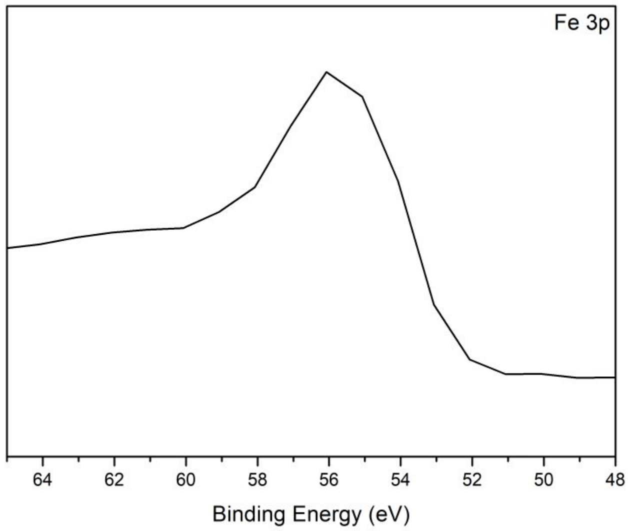

Figure S5: Fe 3p XPS spectrum of SiO_2-pincer catalyst indicating the presence of Fe_2O_3.

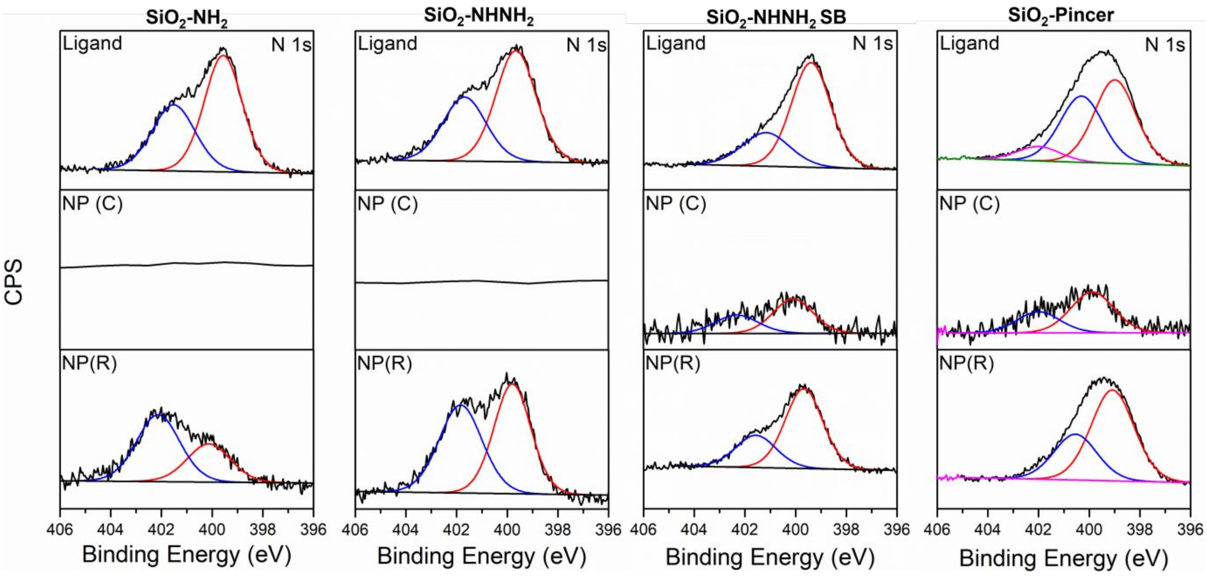

Figure S7: N 1s XPS core level spectra of ligand only and both calcined and chemically reduced NP catalysts for SiO_2-NH_2, SiO_2-NHNH$_2$, SiO_2-NHNH$_2$ SB and SiO_2-pincer ligands.

Table S3. Calculated relative energies (in kcal·mol^{-1}) for the three different spin multiplicities, doublet (D), quadruplet (Q) and sextet (S), for the Fe^{+3} ion using the SiO_2-NH_2 system as a test case.

System	Rel. E. (kcal·mol^{-1})
SiO_2-NH_2-(D)	10.72
SiO_2-NH_2-(Q)	1.76
SiO_2-NH_2-(S)	0.00

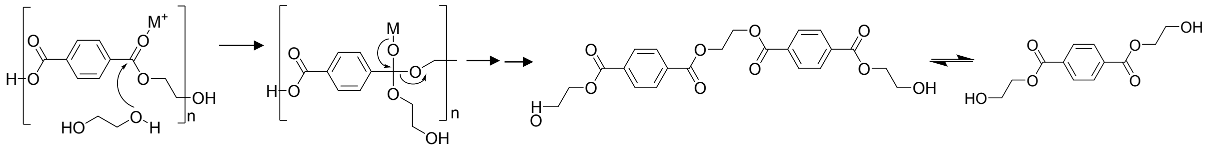

Figure S8: Mechanism for the PET glycolysis reaction.

Table S4: Calculated relative Gibbs energies for the modelled reaction systems, considering 463.15 K and ethylene glycol (EG) as a solvent ($\Delta G_{EG(463.15\ K)}$), including the uncatalyzed process, and the reaction catalyzed by the Fe^{+3} ion, the Fe_2O_3 nanoparticle ($(Fe_2O_3)_2(FeO)_2$) and the FeO nanoparticle ($(Fe_2O_3)(FeO)_{11}$).

System	Uncatalyzed	Fe^{+3}	$(Fe_2O_3)_2(FeO)_2$	$(Fe_2O_3)(FeO)_{11}$
	$\Delta G_{EG(463.15\ K)}$	$\Delta G_{EG(463.15\ K)}$	$\Delta G_{EG(46315\ K)}$	$\Delta G_{EG(463.15\ K)}$
A	0.00	0.00	0.00	0.00
TS_{A-B}	14.95	3.09	1.20	1.35
C	11.58	-3.04	-4.57	-16.20

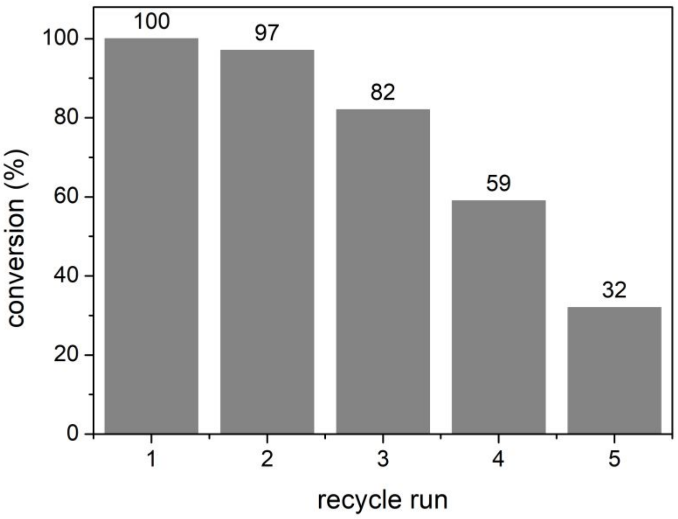

Figure S9: Catalyst recyclability of the SiO_2-NP pincer catalyst over five reaction cycles.

References

1. Bohre, A.; Jadhao, P. R.; Tripathi, K.; Pant, K. K.; Likozar, B.; Saha, B., Chemical Recycling Processes of Waste Polyethylene Terephthalate Using Solid Catalysts. *ChemSusChem* **2023,** *16* (14).
2. Alzuhairi, M. A. H.; Khalil, B. I.; Hadi, R. S., Nano ZnO Catalyst for Chemical Recycling of Polyethylene terephthalate (PET). *Engineering and Technology Journal* **2017,** *35* (8), 831-837.
3. Imran, M.; Kim, D. H.; Al-Masry, W. A.; Mahmood, A.; Hassan, A.; Haider, S.; Ramay, S. M., Manganese-, cobalt-, and zinc-based mixed-oxide spinels as novel catalysts for the chemical recycling of poly(ethylene terephthalate) via glycolysis. *Polymer Degradation and Stability* **2013,** *98* (4), 904-915.
4. Veregue, F. R.; Pereira da Silva, C. T.; Moisés, M. P.; Meneguin, J. G.; Guilherme, M. R.; Arroyo, P. A.; Favaro, S. L.; Radovanovic, E.; Girotto, E. M.; Rinaldi, A. W., Ultrasmall Cobalt

Nanoparticles as a Catalyst for PET Glycolysis: A Green Protocol for Pure Hydroxyethyl Terephthalate Precipitation without Water. *ACS Sustainable Chemistry & Engineering* **2018,** *6* (9), 12017-12024.
5. Fuentes, C. A.; Gallegos, M. V.; García, J. R.; Sambeth, J.; Peluso, M. A., Catalytic Glycolysis of Poly(ethylene terephthalate) Using Zinc and Cobalt Oxides Recycled from Spent Batteries. *Waste and Biomass Valorization* **2020,** *11* (9), 4991-5001.
6. Bartolome, L.; Imran, M.; Lee, K. G.; Sangalang, A.; Ahn, J. K.; Kim, D. H., Superparamagnetic γ-Fe ₂ O ₃ nanoparticles as an easily recoverable catalyst for the chemical recycling of PET. *Green Chem.* **2014,** *16* (1), 279-286.
7. Jeong, J.-M.; Jin, S. B.; Son, S. G.; Suh, H.; Moon, J.-M.; Choi, B. G., Fast and facile synthesis of two-dimensional Fe ^{III} nanosheets based on fluid-shear exfoliation for highly catalytic glycolysis of poly(ethylene terephthalate). *Reaction Chemistry & Engineering* **2021,** *6* (2), 297-303.
8. Al-Sabagh, A. M.; Yehia, F. Z.; Harding, D. R. K.; Eshaq, G.; ElMetwally, A. E., Fe ₃ O ₄ -boosted MWCNT as an efficient sustainable catalyst for PET glycolysis. *Green Chemistry* **2016,** *18* (14), 3997-4003.
9. Lima, G. R.; Monteiro, W. F.; Ligabue, R.; Santana, R. M. C., Titanate Nanotubes as New Nanostrutured Catalyst for Depolymerization of PET by Glycolysis Reaction. *Materials Research* **2017,** *20* (suppl 2), 588-595.
10. Lima, G. R.; Monteiro, W. F.; Toledo, B. O.; Ligabue, R. A.; Santana, R. M. C., Titanate Nanotubes Modified With Zinc and Its Application in Post-Consumer PET Depolymerization. *Macromolecular Symposia* **2019,** *383* (1), 1800008-1800008.
11. Park, G.; Bartolome, L.; Lee, K. G.; Lee, S. J.; Kim, D. H.; Park, T. J., One-step sonochemical synthesis of a graphene oxide–manganese oxide nanocomposite for catalytic glycolysis of poly(ethylene terephthalate). *Nanoscale* **2012,** *4* (13), 3879-3879.
12. Nabid, M. R.; Bide, Y.; Fereidouni, N.; Etemadi, B., Maghemite/nitrogen-doped graphene hybrid material as a reusable bifunctional catalyst for glycolysis of polyethylene terephthalate. *Polymer Degradation and Stability* **2017,** *144*, 434-441.
13. Jeong, J. M.; Jin, S. B.; Park, H. J.; Park, S. H.; Jeon, H.; Suh, H.; Park, Y. J.; Seo, D.; Hwang, S. Y.; Kim, D. H.; Choi, B. G., Large-Scale Fast Fluid Dynamic Processes for the Syntheses of 2D Nanohybrids of Metal Nanoparticle-Deposited Boron Nitride Nanosheet and Their Glycolysis of Poly(ethylene terephthalate). *Advanced Materials Interfaces* **2020,** *7* (16), 2000599-2000599.
14. Cano, I.; Martin, C.; Fernandes, J. A.; Lodge, R. W.; Dupont, J.; Casado-Carmona, F. A.; Lucena, R.; Cardenas, S.; Sans, V.; de Pedro, I., Paramagnetic ionic liquid-coated SiO2@Fe3O4 nanoparticles—The next generation of magnetically recoverable nanocatalysts applied in the glycolysis of PET. *Applied Catalysis B: Environmental* **2020,** *260*, 118110-118110.
15. Najafi-Shoa, S.; Barikani, M.; Ehsani, M.; Ghaffari, M., Cobalt-based ionic liquid grafted on graphene as a heterogeneous catalyst for poly (ethylene terephthalate) glycolysis. *Polymer Degradation and Stability* **2021,** *192*, 109691-109691.
16. Chen, F.; Wang, G.; Li, W.; Yang, F., Glycolysis of Poly(ethylene terephthalate) over Mg–Al Mixed Oxides Catalysts Derived from Hydrotalcites. *Industrial & Engineering Chemistry Research* **2013,** *52* (2), 565-571.
17. Guo, Z.; Adolfsson, E.; Tam, P. L., Nanostructured micro particles as a low-cost and sustainable catalyst in the recycling of PET fiber waste by the glycolysis method. *Waste Management* **2021,** *126*, 559-566.
18. Chen, F.; Yang, F.; Wang, G.; Li, W., Calcined Zn/Al hydrotalcites as solid base catalysts for glycolysis of poly(ethylene terephthalate). *Journal of Applied Polymer Science* **2014,** *131* (22), n/a-n/a.
19. Eshaq, G.; ElMetwally, A. E., (Mg–Zn)–Al layered double hydroxide as a regenerable catalyst for the catalytic glycolysis of polyethylene terephthalate. *Journal of Molecular Liquids* **2016,** *214*, 1-6.
20. Zhu, M.; Li, S.; Li, Z.; Lu, X.; Zhang, S., Investigation of solid catalysts for glycolysis of polyethylene terephthalate. *Chemical Engineering Journal* **2012,** *185-186*, 168-177.

21. Zhu, M.; Li, Z.; Wang, Q.; Zhou, X.; Lu, X., Characterization of Solid Acid Catalysts and Their Reactivity in the Glycolysis of Poly(ethylene terephthalate). *Industrial & Engineering Chemistry Research* **2012,** *51* (36), 11659-11666.
22. Wang, T.; Zheng, Y.; Yu, G.; Chen, X., Glycolysis of polyethylene terephthalate: Magnetic nanoparticle CoFe2O4 catalyst modified using ionic liquid as surfactant. *European Polymer Journal* **2021,** *155*, 110590-110590.
23. Li, M.; Huang, Y.; Yu, T.; Chen, S.; Ju, A.; Ge, M., Chemical recycling of waste poly(ethylene terephthalate) fibers into azo disperse dyestuffs. *RSC Advances* **2014,** *4* (87), 46476-46480.
24. Fung, B. M.; Khitrin, A. K.; Ermolaev, K., An Improved Broadband Decoupling Sequence for Liquid Crystals and Solids. *Journal of Magnetic Resonance* **2000,** *142* (1), 97-101.
25. Stejskal, E. O.; Schaefer, J.; Waugh, J. S., Magic-angle spinning and polarization transfer in proton-enhanced NMR. *Journal of Magnetic Resonance (1969)* **1977,** *28* (1), 105-112.
26. Meier, B. H., Cross polarization under fast magic angle spinning: thermodynamical considerations. *Chemical Physics Letters* **1992,** *188* (3), 201-207.
27. van Meerten, S. G. J.; Franssen, W. M. J.; Kentgens, A. P. M., ssNake: A cross-platform open-source NMR data processing and fitting application. *Journal of Magnetic Resonance* **2019,** *301*, 56-66.
28. Shin, J. H.; Schoenfisch, M. H., Inorganic/Organic Hybrid Silica Nanoparticles as a Nitric Oxide Delivery Scaffold. *Chemistry of materials : a publication of the American Chemical Society* **2008,** *20* (1), 239-249.
29. Hutter, J.; Iannuzzi, M.; Schiffmann, F.; VandeVondele, J., cp2k: atomistic simulations of condensed matter systems. *WIREs Computational Molecular Science* **2014,** *4* (1), 15-25.
30. Ugliengo, P.; Sodupe, M.; Musso, F.; Bush, I. J.; Orlando, R.; Dovesi, R., Realistic Models of Hydroxylated Amorphous Silica Surfaces and MCM-41 Mesoporous Material Simulated by Large-scale Periodic B3LYP Calculations. *Advanced Materials* **2008,** *20* (23), 4579-4583.
31. Perdew, J. P.; Ruzsinszky, A.; Csonka, G. I.; Vydrov, O. A.; Scuseria, G. E.; Constantin, L. A.; Zhou, X.; Burke, K., Restoring the Density-Gradient Expansion for Exchange in Solids and Surfaces. *Physical Review Letters* **2008,** *100* (13), 136406.
32. Grimme, S.; Antony, J.; Ehrlich, S.; Krieg, H., A consistent and accurate ab initio parametrization of density functional dispersion correction (DFT-D) for the 94 elements H-Pu. *The Journal of Chemical Physics* **2010,** *132* (15), 154104.
33. Goedecker, S.; Teter, M.; Hutter, J., Separable dual-space Gaussian pseudopotentials. *Physical Review B* **1996,** *54* (3), 1703-1710.
34. Lippert, B. G.; Parrinello, J. H.; Michele, A hybrid Gaussian and plane wave density functional scheme. *Molecular Physics* **1997,** *92* (3), 477-488.
35. Andreussi, O.; Dabo, I.; Marzari, N., Revised self-consistent continuum solvation in electronic-structure calculations. *J Chem Phys* **2012,** *136* (6), 064102.
